# Isolation and molecular characterization of novel glucarpidases: Enzymes to improve the antibody directed enzyme pro-drug therapy for cancer treatment

**DOI:** 10.1371/journal.pone.0196254

**Published:** 2018-04-26

**Authors:** Fatma B. Rashidi, Alanod D. AlQhatani, Sara S. Bashraheel, Shabnam Shaabani, Matthew R. Groves, Alexander Dömling, Sayed K. Goda

**Affiliations:** 1 Cairo University, Faculty of Science, Giza, Egypt; 2 Anti-doping Lab-Qatar, Research Department, Protein Engineering unit, Doha, Qatar; 3 Drug Design Group, Department of Pharmacy, University of Groningen, Antonius Deusinglaan, AV Groningen, The Netherlands; University of South Alabama Mitchell Cancer Institute, UNITED STATES

## Abstract

Repeated cycles of antibody-directed enzyme pro-drug therapy (ADEPT) and the use of glucarpidase in the detoxification of cytotoxic methotrexate (MTX) are highly desirable during cancer therapy but are hampered by the induced human antibody response to glucarpidase. Novel variants of glucarpidase (formal name: carboxypeptidase G2, CPG2) with epitopes not recognized by the immune system are likely to allow repeated cycles of ADEPT for effective cancer therapy. Towards this aim, over two thousand soil samples were collected and screened for folate hydrolyzing bacteria using folate as the sole carbon source. The work led to the isolation and the characterization of three new glucarpidase producing strains, which were designated as: *Pseudomonas lubricans* strain SF168, *Stenotrophomonas* sp SA and *Xenophilus azovorans* SN213. The *CPG2* genes of *Xenophilus azovorans* SN213 (named *Xen CPG2*) and *Stenotrophomonas sp* SA (named *Sten CPG2*) were cloned and molecularly characterized. Both Xen CPG2 and Sten CPG2 share very close amino acid sequences (99%); we therefore, focused on the study of Xen CPG2. Finally, we demonstrated that a polyclonal antibody raised against our new CPG2, Xen CPG2, does not react with the CPG2 from *Pseudomonas sp*. strain RS-16 (Ps CPG2) that are currently in clinical use. The two enzymes, therefore could potentially be used consecutively in the ADEPT protocol to minimize the effect of the human antibody response that hampers current treatment with Ps CPG2. The identified novel CPG2 in this study will, therefore, pave the way for safer antibody directed enzyme pro-drug therapy for cancer treatment.

## Introduction

Cells use derivatives of folic acid as essential cofactors that are involved in the synthesis of DNA, RNA, proteins, and phospholipids [1, 2]. Mammals cannot synthesize folic acid and thus need to obtain it from their diets. Dietary folate is then converted to various tetrahydrofolate derivatives via a folate pathway.

There are three main enzymes in the folate pathway that are considered as targets for antifolate drugs: Dihydrofolate reductase (DHFR), thymidylate synthase (TS) and glycinamide ribonucleotide formyltransferase (GARFT). Inhibition of DHFR leads to a deficiency of dTMP which, in turn, results in deficient DNA synthesis, DNA breakdown and cell death. Similarly, inhibition of TS will lead to a deficiency in dTMP and cell death. Direct inhibition of GARFT leads to depletion of purine nucleotides, which also leads to cell death [3].

The synthetic folate analog Methotrexate (MTX) is an essential component of many chemotherapeutic regimes that are used for the treatment of patients with neoplastic diseases and has been in clinical use since 1948 [4]. The cytotoxic action of both MTX and its active metabolites is through the inhibition of DHFR, leading to the inhibition of DNA synthesis, repair, and cellular replication. Malignant cells are actively proliferating tissue and, in general, more sensitive to this cellular interference by MTX. Additionally, MTX has an immunomodulating effect and, hence, is used in the treatment of some other diseases such as rheumatoid arthritis, multiple sclerosis and psoriasis [5, 6].

There are also different analogs for MTT in various stages of preclinical and clinical development [7, 8]. Pemetrexed, a potent polyglutamate classic antifolate TS inhibitor formerly called LY23154 [9], is an interesting new antifolate. The novel structure of Pemetrexed makes it different from other antifolates and, in contrast to MTX, Pemetrexed can inhibit several enzymes that are involved in the synthesis of purines and pyrimidines. Pemetrexed, similarly to MTX and other antifolates, has an unacceptable level of toxicity, creating major drawbacks in clinical use [10].

The ability to degrade antifolate drugs rapidly in vivo would have major clinical advantages. It would minimize toxicity, allowing a higher dose of antifolate compounds to be administered, potentially leading to a higher clinical efficacy.Enzymatic degradation to remove the excess of these drugs is considered a highly effective mechanism and can be achieved through the therapeutic use of glucarpidases [10].

Glucarpidase (also known as carboxypeptidase G2 or CPG2, Ps CPG2) is produced from *Variovorax paradoxus* (*Pseudomonas sp*. strain RS-16), it has no mammalian equivalent and is a zinc-dependent dimeric protein composed of two subunits of 41kDa [11, 12]. Glucarpidase has a relatively restricted substrate specificity and hydrolyzes the C-terminal glutamic acid residue of folic acid, poly-glutamyl derivatives of folic acid and folate analogs, including methotrexate and sub-fragments of folic acid, e.g., *p*-aminobenzoyl glutamate [13] to yield pteroate derivatives. Glucarpidase converts methotrexate into a less toxic product, pteroate, leading to a rapid decrease in MTX levels in the serum in both animal models and in humans. Accordingly, the enzyme is a powerful rescue agent in patients receiving toxic doses of MTX, and glucarpidase treatment might be clinically attractive for MTX dose escalation studies [4].

Another important medical application of glucarpidase is its use in targeted cancer therapy. There are two major obstacles in systemic cancer therapies: a lack of tumor selectivity and drug resistance. Antibody Directed Enzyme Pro-drug Therapy (ADEPT) [14–18] is a therapy that solves these two problems by generating a powerful agent in the vicinity of the tumor site. ADEPT has been successfully used in animal tumor models of human choriocarcinoma, as well as colonic and breast carcinoma. Glucarpidase is the most widely used enzyme in the ADEPT-based clinical trials [19–22]. The activity of glucarpidase can be exploited to cleave glutamic acid moieties of a variety of pro-drugs to release potent nitrogen mustards. Of these, 4-[(2-chloroethyl)(2-mesyloxyethyl)amino-]benzoyl-L-glutamic acid (CMDA), is cleaved by Ps CPG2 to a cytotoxic agent, 4-[(2-chloroethyl)(2-mesyloxyethyl)amino benzoic acid. Additionally, a variety of glucarpidase-antibody chemical conjugates targeting CPG2 activity to tumors have been evaluated [23]. However, treatment with glucarpidase results in a severe immune response following repeated use, limiting its therapeutic applicability. Thus, new CPG2s that differ in their immunogenicity relative to Ps CPG2 would increase the chances of success in repeated cycles of ADEPT by triggering a lower human antibody response.

To address this problem, we screened for new glucarpidase-producing bacteria from soils followed by the isolation, cloning and overexpression of their novel glucarpidase(s) for functional investigation and characterization.

## Materials and methods

### Growth media, enzymes, chemicals and antibodies

Folate minimal medium (FMM) contains per liter: 3g KH_2_PO_4_, 6g Na_2_HPO_4_ (anhydrous), 1g NaCl, 0.13g MgSO_4_.7H_2_O and 0.01g FeSO_4_.7H_2_O. All salts were dissolved in distilled water, the pH of the solution was adjusted to 7.2 and the volume was adjusted to 1 liter with further distilled water prior to autoclaving at 121°C at 15 p.s.i. for 15 min; filter-sterilized 0.5% (v/v) folic acid (Sigma) was then added as the sole carbon source. The medium was solidified with 1.5% (w/v) Bacto-agar when necessary.

Enzymes for cloning and expression of the glucarpidase genes were purchased from Thermo Scientific, with the exception of *Sau*3AI, *Bam*H1 and a PCR master mix (2x) kit, which were purchased from Promega. Ni-NTA resin was purchased from Sigma. GelPilot 1 kb DNA ladder (100) was purchased from Thermo Scientific, Wizard® SV Gel and PCR Clean-Up System Kit were purchased from Promega. GeneJET Plasmid Miniprep Kit was obtained from Thermo Scientific. All other chemicals were of a high analytical grade.

The Mass spectroscopy (MS) analysis was carried out at the Toxicology and Multipurpose Labs, ADL-Qatar. Anti-Xen CPG2 Polyclonal Antibodies were produced by Eurogentec, Belgium, anti-Rabbit IgG (whole molecule)–Peroxidase antibody produced in goat (Sigma) Catalog No. 40545 was used as the secondary conjugated antibody. 6x His Epitope Tag Antibody (HIS. H8) (Thermo Scientific) Catalog No. MA1-21315 was used for detection of the purified 6 His tagged CPG2 and Polyclonal Rabbit Anti-Mouse Immunoglobulins/HRP (Dako) ref. code P0260 was used as secondary antibody.

### Isolation of glucarpidase producing bacteria

#### Bacterial sample collection

More than two thousands soil samples were collected from the surface layer (0–15 cm) of thirty different agricultural sites in Egypt (Giza, Berquash and Manshaet Alqanater) and Qatar (Abu-Breaka, Aljamel, Alshamal). The samples were collected from private agricultural lands after the permission of the owners in both countries. The field studies did not involve endangered or protected species. These fields were previously cultivated with fruits and vegetables rich in folates such as oranges, asparagus and broccoli.

#### Isolation of folate hydrolyzing bacteria by an enrichment technique

After thorough mixing, preparations were incubated at 37°C for five days in 5 mL growth media to increase the probability of identifying folate-hydrolyzing bacteria. Subsequently, 1 mL was used to inoculate 50 mL autoclaved M9 minimal salt medium (MSM) [24] containing folate (0.5%) as the sole carbon source. Cultures were then incubated in an orbital shaker for three days at 37°C (close to the weather temperature where the soils were collected) and 160 rpm. After three days, cultures were allowed to settle for one hour, and 1 mL of each supernatant was used to inoculate another 9 mL of fresh enrichment medium prior to incubation for an additional five days under the same conditions. After three subsequent enrichments in the same media, cultures were serially diluted (10-4-10-8) and plated on the same media. Plates were incubated at 37°C until bacterial colonies became visible. Selected isolates were stored at 4°C.

#### Identification of isolated bacterial strains by *16S rDNA*

Three isolates—NS1 and FS3 and AS1—were subsequently selected for further molecular characterization and identification by Vitek 2 Compact (GN kit) from bioMérieux, PCR amplification and partial sequence analysis of their *16S rRNA* genes.

A single colony of each strain was re-suspended in 1 mL of PCR-grade water and boiled for 10 min. The diluted lysate was used as a template for PCR amplification of *16S rDNA* using primer pair 27F (5' AGAGTTTGATCATGGCTCAG 3') and 1492R (5' CGGTTACCTTGTTACGACTT 3'). PCR was carried out using a GeneAmp PCR System 9700 (Applied Biosystems, Foster City, CA, USA) with the following amplification conditions: initial denaturation at 94°C for 5 min, 35 cycles of 30 sec denaturation at 94°C, 30 sec annealing at 60°C and 1 min extension at 72°C, followed by a final extension at 72°C for 10 min. The PCR product was cloned into pGEM-T vector (Promega, UK) following the manufacturer’s instructions and transformed into *E*. *coli* JM109 competent cells. Following isolation of the transformants, plasmid DNA was purified and sequenced. *16S rDNA* sequence of each strain was submitted to a BLAST search of the NCBI (National Center for Biotechnology Information) GenBank database (www.blast.ddbj.nig.ac.jp/) to identify the organism.

### Native glucarpidase activity assay

#### Determination of glucarpidase activity in the three identified strains

Cells of strains NS1, FS3 and AS1 were sonicated in 0.1 M Tris-hydrochloride (pH 7.3) containing 0.2 mM ZnSO_4_, and the soluble fraction tested for glucarpidase activity. Glucarpidase activity was determined using methotrexate (MTX) as the substrate by the modification of the method of McCullough [25]. 590 µl of 0.1 M Tris-HCl pH 7.3 containing 0.2 mM ZnSO_4_ and 5 µl of MTX (0.45 mM) was equilibrated at 37°C for 10 minutes, then the total protein extract of each strain (50 µg/mL) was added and incubated at 37°C. Samples were taken at 10 min intervals, and the decrease in absorbance at 320 nm was measured using a NANODROP 1000 spectrophotometer (Thermo Scientific). The same protocol was performed to analyze the activity of the pure recombinant CPG2 using 3 µg/mL protein. The obtained data was plotted using GraphPad Prism6.

#### Effect of zinc ion on glucarpidase activity

A 1-mL reaction cuvette containing 0.1 mL of 0.45 mM MTX and 0.9 mL of 0.1 M Tris-hydrochloride (pH 7.3) in the presence of 0.2 mM ZnSO_4_ was equilibrated at 37°C. Protein extract, 20 µl of 1 mg/mL, was added and the decrease in absorbance at 320 nm was measured with a Pye-Unicam SP1800 double-beam spectrophotometer. The same experiment also was done in the presence of 0.5 mM EDTA.

### New *Xen* and *Sten CPG2* gene(s) isolation and cloning into vector pET28a

#### Genomic DNA library construction and screening

Genomic DNA from *Xenophilus sp*. SN213 and *Stenotrophomonas sp* AS was isolated using a DNA extraction kit (Thermo Scientific), partially digested by *Sau*3AI, and fragments in the range of 2–4 kb were isolated by gel electrophoresis. The resulting fragments were cloned into *Bam*H1-digested pET28a, (pET28a has been used successfully in our laboratory for cloning and overexpression of many genes) and then transformed into competent cells of *E*. *coli* BL21 (DE3) RIL. The transformants were screened on LB-Kan-folate-IPTG plates (containing 32 μg/mL kanamycin, 0.5% (v/v) folate and 0.1% IPTG). Positive clones of yellow color were picked and plasmids isolated as described above.

#### Sequence analysis, isolation, and cloning of the Xen CPG2 and Sten CPG2

The selected constructs were sequenced using universal primers T7 promoter and terminator. ORF searches and sequence analysis were performed using the DNASTAR program and the obtained sequences were subjected to homology search using the National Centre for Biotechnological Information (NCBI) on-line program BLAST. Sequence comparison was performed using pairwise and multiple sequence alignment program CLUSTAL W [26, 27]. Primers were designed based on the ORF of the two new CPG2, and unique restriction enzymes (*Nde*I/*Hind*III) were introduced to facilitate the cloning of the gene(s).

*CPG2*-Fwd 5' ACC GGA TCC CAT ATG CAG AAG CGC GAC AAC GTG CTG TTC C 3' (the underlined sequence is *Nde*I site).

*CPG2*-Rev 5' CCT AAG CTT TCA CTT GCC GGC GCC CAG ATC CAT G 3' (the underlined sequence is *Hind*III site).

The PCR program used for amplification was 94°C for 5 min (1 cycle), followed by 35 cycles of 94°C denaturation for 1 min, 64°C annealing for 1 min and 72°C extension for 1.5 min, followed by a final extension at 72°C for 10 min (1 cycle). The purified PCR product was digested with *Nde*I and *Hind*III and ligated into similarly digested, dephosphorylated pure pET28a DNA using T4 DNA ligase.

The ligated mixtures were transformed into chemically competent *E*. *coli* and spread over LB-agar plates supplemented with 32 μg/mL kanamycin and incubated at 37°C overnight. Plasmid DNA was extracted from randomly selected colonies, following the manufacturer’s (Thermo Scientific) recommended protocol. Miniplasmid preparations were checked for the presence of the gene of interest by double restriction digestion reaction using *Nde*I/*Hind*III. Finally, the constructs of both new *CPG2*, *Xen CPG2* and *Sten CPG2* were confirmed by DNA sequencing using universal T7 promoter and terminator primers.

### Xen CPG2 protein expression and recombinant protein purification

#### Protein expression

The pET28a-Xen CPG2 was transformed into the expression host *E*. *coli* BL21(DE3)RIL according to the manufacturer’s instructions (Stratagene) and transformants were selected on LB-agar plates supplemented with Kanamycin (Kan) and Chloramphenicol (Cam) at final concentration of 32 µg/mL each at 37°C. A single colony was incubated in 10 mL LB medium supplemented with the required antibiotics at 37°C. 50 mL of fresh autoclaved LB-broth supplemented by the corresponding antibiotics was inoculated with 250 µl of the overnight culture. The culture was incubated at 37°C in an incubator shaker until the optical density at 600nm reached 0.5–0.6. Induction of recombinant CPG2 was initiated by the addition of isopropyl-β-D-thiogalactopyranoside (IPTG) at a final concentration of 1 mM then incubated for further four hours at 37°C with shaking at 200 rpm. Cells were collected by centrifugation at 4000rpm for 20 min at 4°C. Cell pellets were re-suspended in 20 mM Tris buffer pH 7.5 containing 50 mM NaCl and then were disrupted by sonication on ice, 5 cycles of 30 sec sonication pulses followed by I min rest. The soluble fraction was separated by centrifugation at 14,000 rpm for 20 min at 4°C. The soluble and insoluble fractions were mixed with 2X sample buffer and boiled for 10 minutes at 95°C, then applied to SDS-PAGE analysis. Protein expression was performed, where the induction step with IPTG at 20°C was carried out identically to assess the effect of temperature to improve soluble protein expression. The CPG2 expressed in the soluble form was used for all our studies. We did not carry out refolding of the inclusion bodies.

#### Ni-NTA purification

To facilitate the purification of Xen CPG2, the gene was sub-cloned into the *Nde*I/*Hind*III sites of pET-28a, creating a gene that encoded an *N*-terminal His_6_ tag. The soluble protein was subjected to purification by Ni2+ affinity chromatography using Ni-NTA resin. About 1 mL of the resin was washed with distilled water and activated by binding and washing buffer A (20 mM Tris pH 8, 50 mM NaCl, 5 mM BME, and 20 mM imidazole), then the total soluble protein was combined with the activated resin and gently agitated for 20 min at 4°C to allow the protein to bind to the column resin. The resin was separated by gravity, the flow-through was collected, and the resin was washed 3 times with buffer A. The target protein (bound to the resin) was collected by adding ice-cold elution buffer B (20 mM Tris pH 8, 50 mM NaCl, 5 mM BME and 400 mM imidazole). The eluted protein was dialyzed against 100 mM Tris-HCl pH 7.3 containing 0.2 mM ZnSO_4_. All fractions of the protein purification were analyzed by SDS-PAGE analysis. Hydrolytic activity of the pure recombinant glucarpidase (at conc. 3 μg/mL) was assayed spectrophotometrically using MTX (substrate) as described above.

#### *Pseudomonas sp*. strain RS-16, Ps CPG2 protein expression and purification

A synthetic gene for *Pseudomonas* CPG2 codon-optimized for maximum expression in *E*. *coli* B21(DE)3RIL[28], was cloned, overexpressed and purified following the same procedures used for Xen CPG2.

#### MS of folate degradation by Xen and Ps CPG2

The insoluble materials formed from folate degradation by Xen CPG2 or Ps CPG2 containing bacteria were collected and dissolved in NaOH pH 9.

3 µl aliquot of each sample was injected onto a 2.1 x 50 mm Acquity BEH C18 (1.7 um particle size) UPLC column using the following eluents: A = water + 0.1% formic acid and B = acetonitrile + 0.1% formic acid. Eluent flow was 300 µl/min operating in an isocratic mode consisting of 7% B. The column was thermostatted at 40°C. The UHPLC was a Dionex UniMate 3000 Binary RSLC (Thermo Fisher Scientific).

Eluent from the UPLC column was passed to a HESI II electrospray ion source operating in the positive ion mode. Data were acquired on a QExactive Classic HRAM orbitrap mass spectrometer (Thermo Fisher Scientific) in full scan high resolution (70,000 mass resolution) accurate mass mode (HRAM). Accurate mass results were acquired with external calibration. Protonated parent ion current profiles for the targeted parent masses were extracted with a mass tolerance of +/- 5 ppm. Background subtracted spectra showed protonated parent masses with a mass accuracy of better than 3 ppm (external calibration).

### Kinetics properties of Xen CPG2 and Ps CPG2

Methotrexate was used for determination of the Michaelis constant (K_m_) and the rate of reaction (V_max_). Both purified enzymes, Xen CPG2 and Ps CPG2 (2.12 µg/mL), were assayed at various methotrexate concentration (0.03 up to 0.42 mM) in 0.1 M Tris-HCl pH 7.3 and 0.2 mM ZnSO_4_ using Nunc 96 plates with UV transparent flat bottom wells. All reactions were carried out at 37°C for 2 min and the decrease in absorbance at 320 nm was determined using Infinite M200 PRO NanoQuant Plate Reader (TECAN). Apparent K_m_, V_max_ and K_cat_ values of each protein were determined by fitting to the Michaelis-Menten equation using GraphPad PRISM 6 software.

One unit of the enzyme represents the hydrolysis of 1mM of MTX per min at 37°C. The enzyme activity per ml of extract was calculated by using an extinction coefficient for MTX of 8,300.

### Biophysical characterization of Xen CPG2 by Circular dichroism

#### Sample preparation

Pure Xen CPG2 was re-dialyzed against sterile milli-Q water; then the protein was centrifuged 30 min. at 4°C to get rid of any aggregates. The protein concentration was measured using Nanodrop 2000 spectrophotometer (Thermo Scientific) and adjusted to about 0.5 mg/mL with water. The extinction coefficient for glucarpidase was taken as

*ε* = 23380 M-1 cm-1 for Xen CPG2 and 24870 M-1 cm-1 for Ps CPG2.

#### Circular dichroism (CD)

All CD experiments were carried out at 20°C using a Chirascan™ Plus CD Spectrometer (Applied Photophysics). Protein concentrations of 0.5 mg/mL to 0.6 mg/mL were measured in a SUPRASIL Quartz demountable rectangular (Hellma®) cuvette of 0.2 mm light-path length (sample volume ∼60 μl).The used CD parameters were: bandwidth 1 nm and a scan time per point of 0.5 s. Four average and smoothed scans per sample were measured using the Chirascan analysis software. Additionally, an averaged buffer (water) CD signal over the same wavelength region was determined and used as a buffer baseline, and each averaged CD spectrum was corrected for the buffer baseline by subtraction. The resulting molar ellipticity [θ] was calculated per spectrum by applying the molar protein concentration [M] and the pathlength of the used cuvette (2mm) and following the equation:

MolarEllipticity[θ]=(100×θ)/(Conc×Pathlength)

Where θ is the ellipticity measured in degrees, Conc. is concentration measured in mole/L, the pathlength is measured in cm, and molar ellipticity has units of deg.cm2.dmol^-1^.

#### CD spectra deconvolution method

Protein secondary structure was estimated by CD data deconvolution analysis using the CDNN (version 2.1) software tool to deconvolute the CD data spectra in the far UV spectral region. Deconvolution of the CD data was done in the 190–260 nm spectral region. In the deconvolution calculations, the number of amino acids/residues was taken for Xen CPG2 as 392 AAs, with a molecular weight of 41.76148 kDa and for Ps CPG2; 394 AAs, with 41.91174, protein conc. (0.58 mg/mL) and 0.02 cm light-pathlength of the cuvette was used. Four repeat scans were measured per sample and then averaged.

#### Homology modeling of Xen CPG2

Homology modeling was performed using SWISS-MODEL, a fully automated protein structure homology-modeling server, accessible via the ExPASy web server, or from the program DeepView (Swiss Pdb-Viewer) [29]. Briefly, we searched for templates matching our target Azo CPG2 sequence. The closest enzyme, carboxypeptidase G2 (PDB ID 1CG2) with a sequence homology of 94% was used to build a model. The model quality evaluation was performed using GMQE (Global Model Quality Estimation). The model was visualized using PyMol.

#### Immunoblotting of Xen CPG2 and Ps CPG2 using anti His and anti Xen CPG2 antibodies

Xen CPG2 or Ps CPG2 were separated by SDS-PAGE and transferred to PVDF membranes overnight at 30 V using a Bio-Rad Mini-Trans-Blot tank and Tris-glycine buffer (25 mM Tris, 192 mM glycine, pH 8.3). For detection of each CPG2 protein, a polyclonal anti-(His)_6_ antibody (Sigma) or a polyclonal anti CPG2 were used separately as the primary antibody (1:3000 dilution), with HRP-conjugated ECL anti-rabbit antibody (GE Healthcare, 1:10,000 dilution) used as the secondary antibody. Detection was performed using ECL chemiluminescent detection reagent as described by the supplier (GE Healthcare). In addition, dot blots were carried out at different protein concentrations (0.05, 0.1, and 0.2 mg/mL) and at different anti Xen CPG2 dilutions (1:20 000, 1:10 000 and 1:3000).

## Results

### Isolation and characterization of glucarpidase producing bacterial strains

Seven folate hydrolyzing bacterial strains (NS1-3, FS1-3, and AS1) were successfully isolated using the described enrichment technique. A clear halo appeared around colonies capable of hydrolyzing folate, which indicates the presence of glucarpidase activity in the isolated bacteria. Due to the larger halos formed around NS1, FS3 and AS1, these isolates were chosen for further studies.

The screened isolates NS1, FS3, and AS1 showed growth after two days on minimum folate media (FMM) agar-plate and showed complete degradation of the folate after 5 days (data not shown).

**Fig 1:**
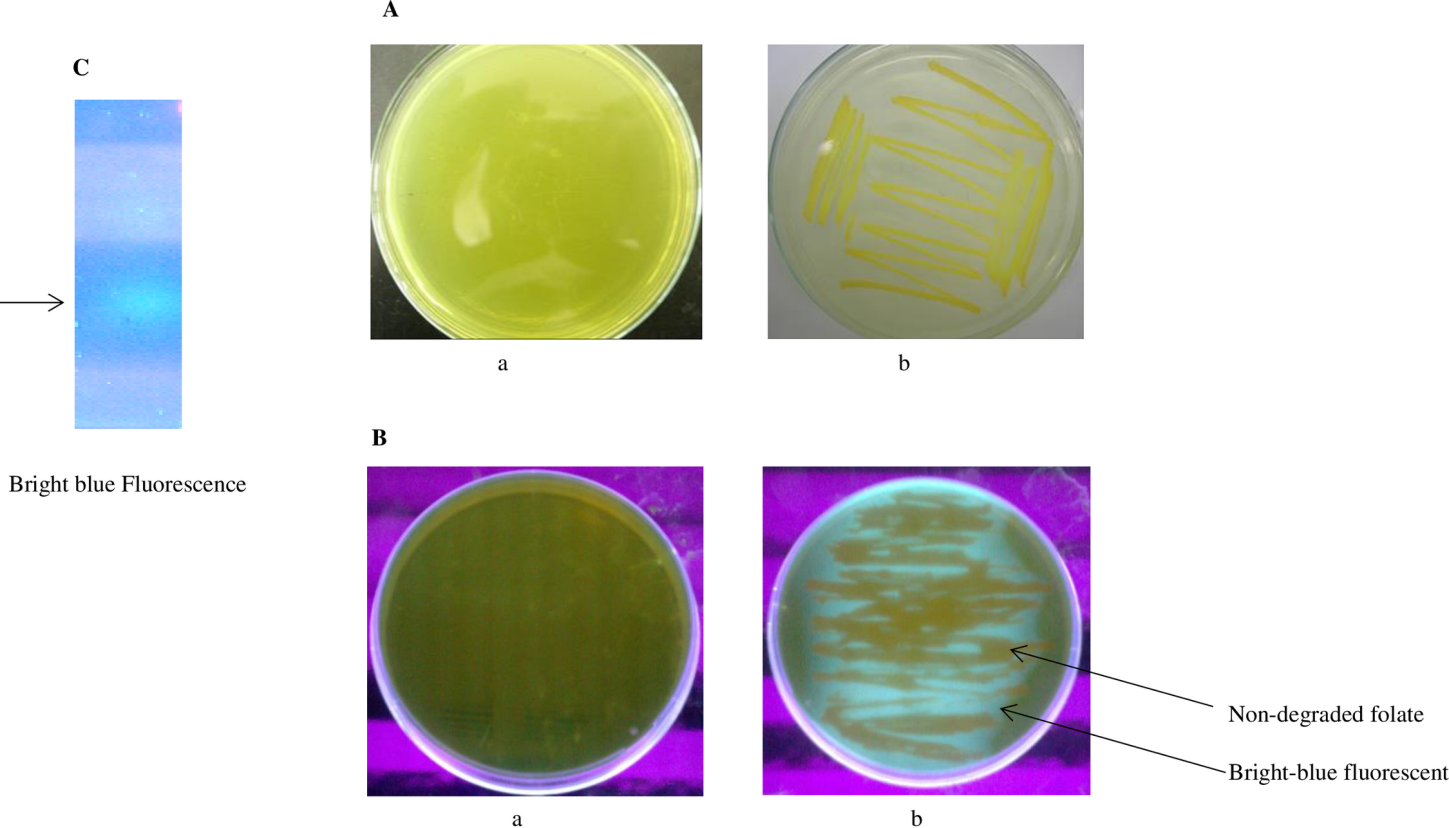
Growth of glucarpidase producing bacteria. **1A**. Bacterial growth on folate as the only carbon source LB/agar plate. (a) Before the bacterial growth and (b) the bacterial growth after several days. **1B**. A bright-blue fluorescence under U.V. light. (a) The Folate media under UV light while (b) is the growing bacteria on folate plate showing the bright-blue fluorescence of lumazine-6-carboxylic acid resulting from folate hydrolysis by the growing strain. **1C**. 1% Agarose gel electrophoresis of the cell extract of one isolate showing bright blue fluorescence (lumazine-6-carboxylic acid) under U.V. light, resulting from folate degradation by the isolated strain.

Fig 1A and 1B shows the colorless areas around the growing bacterial strain FS3 as bright-blue fluorescence under UV light resulting from the accumulation of lumazine-6-carboxylic acid, which was confirmed by analyzing cell extracts by gel electrophoresis (Fig 1C).

**Fig 2:**
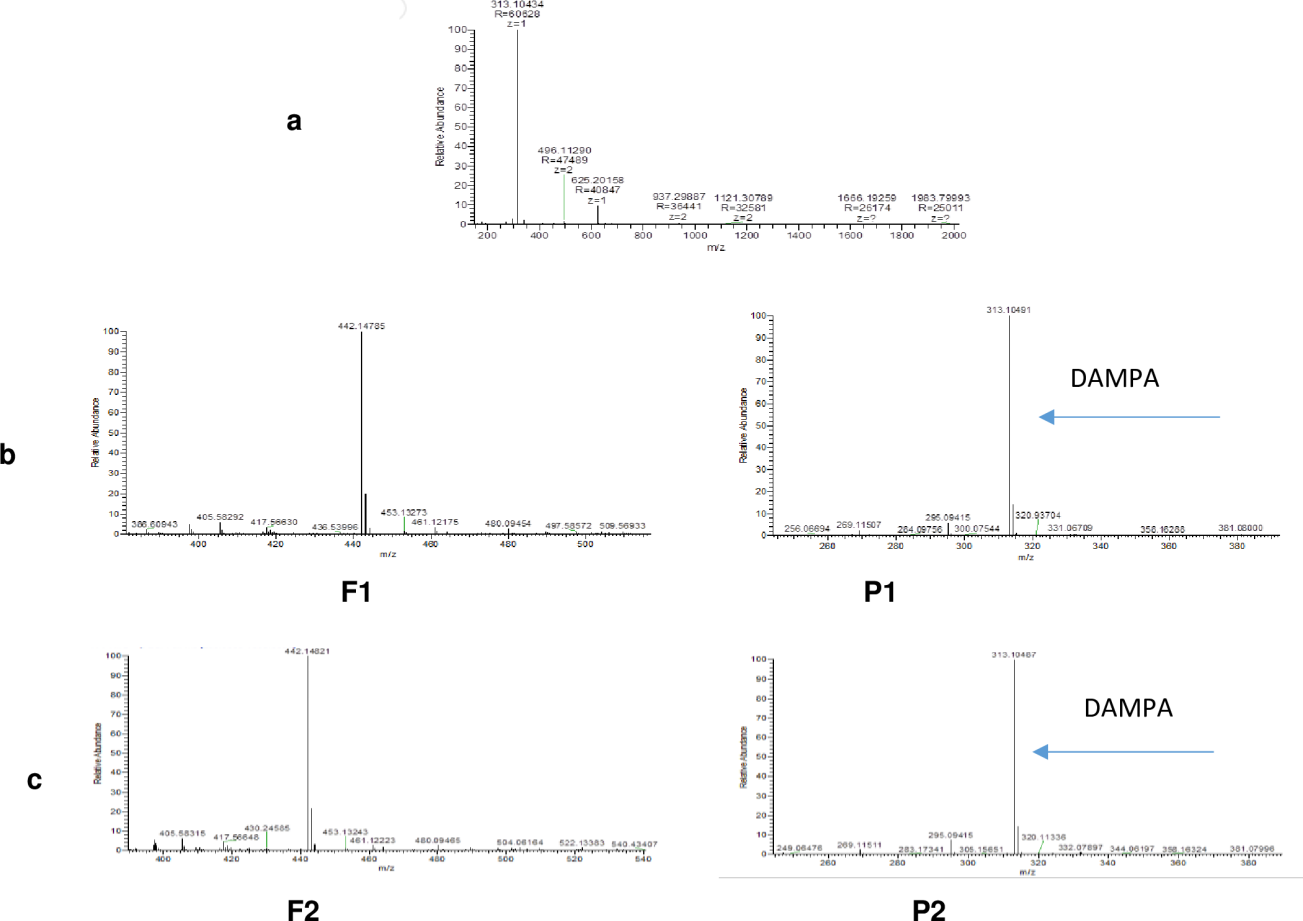
MS of the protonated DAMPA is shown at 313.1m/z. a) DAMPA H+ peak is the product of folate hydrolysis by the isolated strain. b and c) P1, P2 are DMPA H+ produced by recombinant CPG2s, new and Ps CPG2 respectively and F1, F2 are intact folate isolated from the media of both recombinant enzymes.

We also confirmed the hydrolysis of folate by glucarpidase containing strains, and the production of DAMPA (2, 4-diamino-N-10-methyl pteroic acid) the insoluble product by mass-spectroscopy (MS). (Fig 2A) Shows a mass of 313.1 m/z, corresponding to the calculated mass of DAMPA H+ from one strain. Similar results were obtained from the recombinant *E*. *coli* containing glucarpidase(s) (Xen and Ps CPG2) (Fig 2B and 2C).

### Molecular identification of soil isolates: PCR amplification and sequence analysis of *16S rRNA* gene

Results from microbiological identification of the isolate(s) using Vitek 2 Compact and DNA sequence similarity analysis of the PCR products from the *16S rRNA* genes of the NS1, AS1, and FS3 isolates indicated that the 1317 bp of *16S rRNA* gene of NS1 is most closely related to *Xenophilus azovorans* strain KF46F (98%) and was identified as *Xenophilus azovorans* SN213. The results of the sequence similarity analysis indicated that the 1254 bp of *16S rRNA* gene of FS3 was identical to that of *Pseudomonas lubricans* (100%) and *Pseudomonas sp*. PASS-camb (99%). This analysis indicated that strain FS3 belonged to *Pseudomonas* species and was identified as *Pseudomonas lubricans* strain SF168. The AS1 strain is 100% identical to *Stenotrophomonas sp*. AB1 and was named as *Stenotrophomonas sp*. AS. To establish the phylogenetic position of the isolated strain(s), their 16s rRNA sequence(s) were compared with other sequences of their closely related species retrieved from the GenBank database. Their resulting phylogenetic tree based on the Fast Minimum Evolution Method is shown in supplementary data (S1 and S2 Figs).

### GenBank accession numbers

The sequence and the name of the identified strains were then deposited to the NCBI GenBank, (*Pseudomonas lubricans* Strain SF168, accession number FJ600733 and *Xenophilus azovorans* SN213, Accession number EU650684).

### Zinc dependence of the native glucarpidase activity

**Fig 3:**
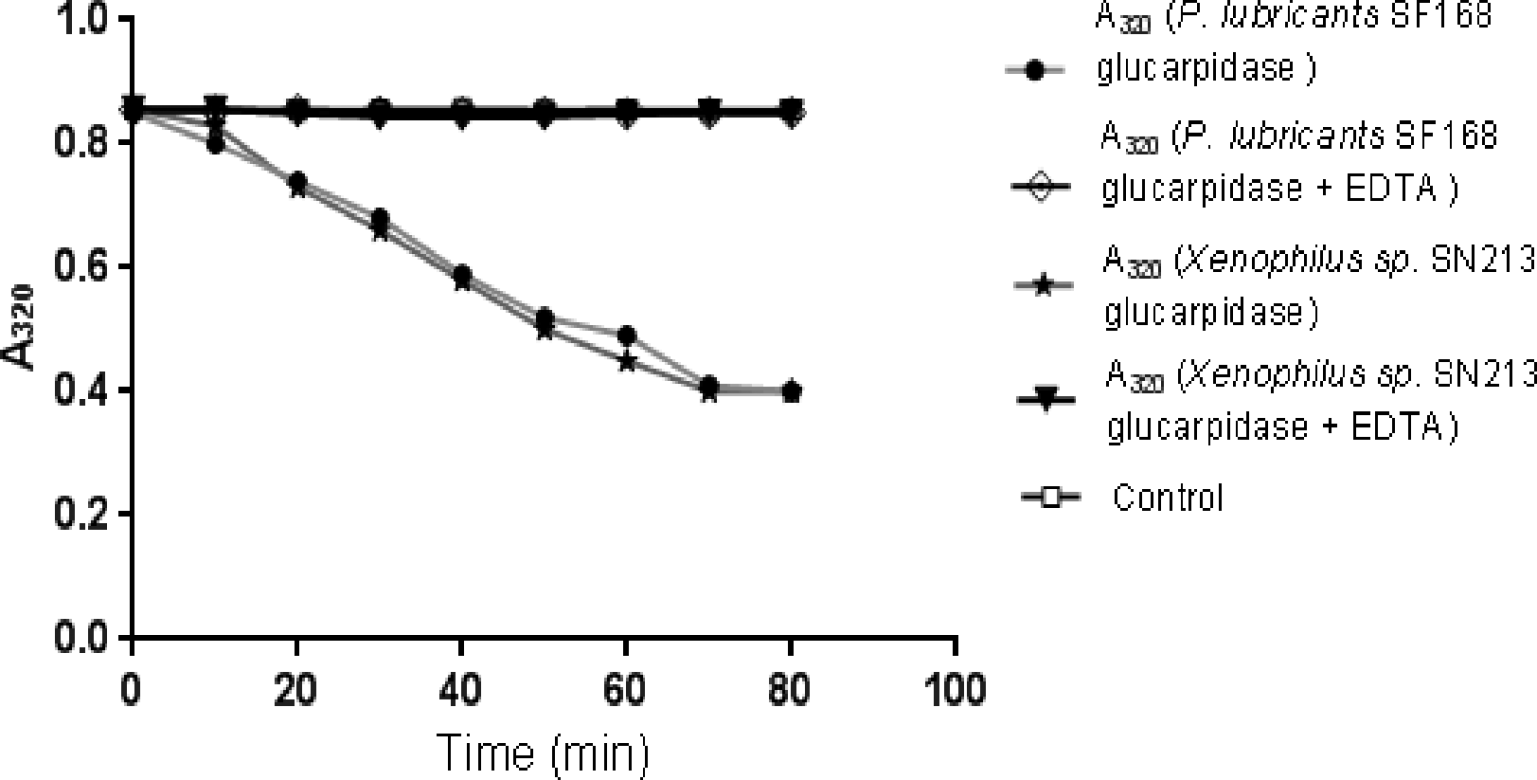
Glucarpidase activity in the total soluble protein of the two novel glucarpidase producing bacteria. MTX substrate solution and total soluble protein of *Pseudomonas lubricants* strain SF168 in presence of Zn^2+^ (filled ball) and in presence of Zn^2+^ and EDTA (diamond). MTX substrate solution and total soluble protein from *Xenophilus* sp. SN213 in the presence of Zn^2+^ (star), and in the presence of Zn^2+^ and EDTA (filled triangle), and control of MTX and enzyme buffer (square) are shown. The data in this figure indicates that both strains (*Pseudomonas lubricans* strain SF168 and *Xenophilus* sp. SN213) show CPG2 activity through methotrexate hydrolysis.

The data in Fig 3 indicate that the total protein extract of *Pseudomonas lubricans* strain SF168 and *Xenophilus azovorans* SN213 showed no glucarpidase activity towards the substrate methotrexate in the absence of Zn2+ ions. However, when Zn2+ was added to the assay mixture, glucarpidase activity could be measured. We carried out the same experiment in the presence of 10 mM EDTA. No enzyme activity was detected in the presence of EDTA.

### Cloning and overexpression of the new glucarpidases from *Xenophilus azovorans* SN213 and *Stenotrophomonas sp*. SA

To clone the *CPG2* genes from the newly isolated strains, chromosomal DNA libraries from both isolated strains were prepared and screened for CPG2 producing colonies using media where folate is the only carbon source. The resultant recombinant plasmids were designated pCPGxen16 and pCPGsten8, for CPG2 from *Xenophilus azovorans* SN213 and *Stenotrophomonas sp*. SA respectively. Determination of the nucleotide sequence of each fragment in both plasmids revealed the amino acid sequences of each recombinant protein. In each case, the identified open reading frame is 1,176 nucleotides long and encodes proteins with 392 amino acids.

Partial nucleotides and amino acid sequence data for CPGxen16 appear in the DDBJ/ GenBank database under accession numbers JX192958 and AGI21725, respectively.

**Fig 4:**
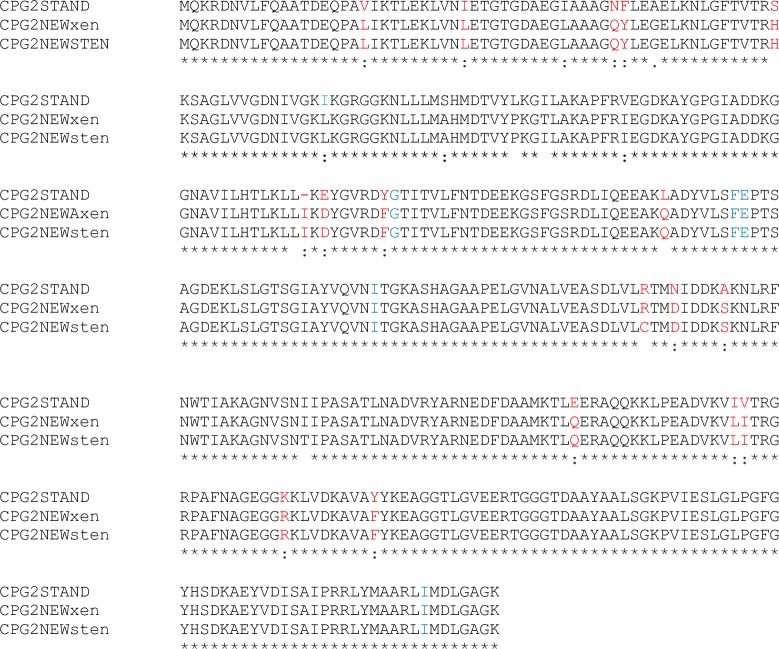
Amino acids sequence alignment of glucarpidase genes. the uppermost sequence encodes the glucarpidase from *Pseudomonas sp*. Strain RS-16 (12), the lower sequences encode the new glucarpidases isolated from *Xenophilus azovorans SN213* and *Stenotrophomonas sp SA*, respectively (this work). Amino acid differences between the sequences are highlighted in red. Amino acids in blue are involved in the active site of the enzyme where two zinc ions and a bridging water molecule binds (30).

The calculated molecular mass of the deduced amino acid sequence of the CPGxen16 was 41761.4 Da, which is in close agreement with that of the native enzyme [12]. A search of the protein database using the program BLAST revealed that the deduced amino acid sequence of the Xen CPG2 exhibits a high identity (94%) to that of CPG2 from the strain of *Pseudomonas sp*. Strain RS-16 [12, 30]. CPG2sten8 exhibits 99% similarity with CPGxen16. Their amino acid sequence alignment is shown in Fig 4.

**Fig 5:**
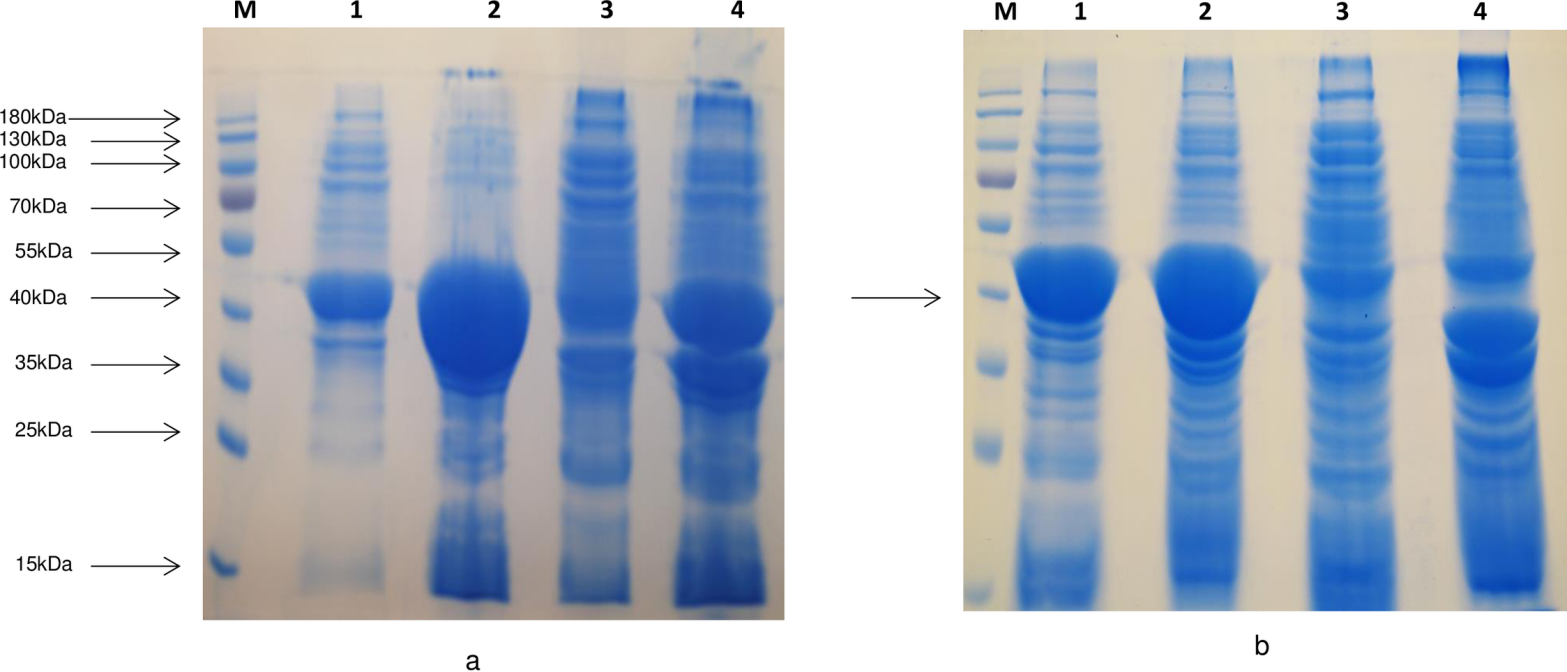
CPG2 Protein expression of the gene isolated from *Xenophilus azovorans* SN213. **a**. Coomassie blue staining of 10% SDS-PAGE of *E*. *coli* BL21(DE3)RIL-CPG2 protein expression at 37°C of the gene isolated from *Xenophilus azovorans* SN213. M is the PageRuler Prestained Protein Ladder (10 to 180 kDa), lanes 1 and 2 are the induced soluble and insoluble fractions respectively, and lanes 3 and 4 are the uninduced soluble and insoluble fractions respectively. **b**. Protein expression at 20°C of the gene isolated from *Xenophilus azovorans*. SN213. M is the prestained protein ladder, lanes 1 and 2 are the induced soluble and insoluble fractions respectively, and lanes 3 and 4 are the uninduced soluble and insoluble fractions respectively.

SDS-PAGE analysis showed that a high level of protein expression (160 mg/L) was observed (Fig 5A). The CPG2s of both new strains were overexpressed but about 80% of the protein was present as inclusion bodies. The expression of soluble CPG2 did not improve by varying the IPTG concentration (data not shown). However, when cells were induced at 20°C for overnight, we found that soluble CPG2 expression was significantly increased (80 mg/L) (Fig 5B). The molecular weight of over-expressed glucarpidase was about 41 kDa, as determined by SDS-PAGE, and is consistent with the calculated molecular mass from the deduced amino acid sequence of the isolated gene.

### Protein purification and activity assay of the newly isolated glucarpidase, Xen CPG2

**Fig 6:**
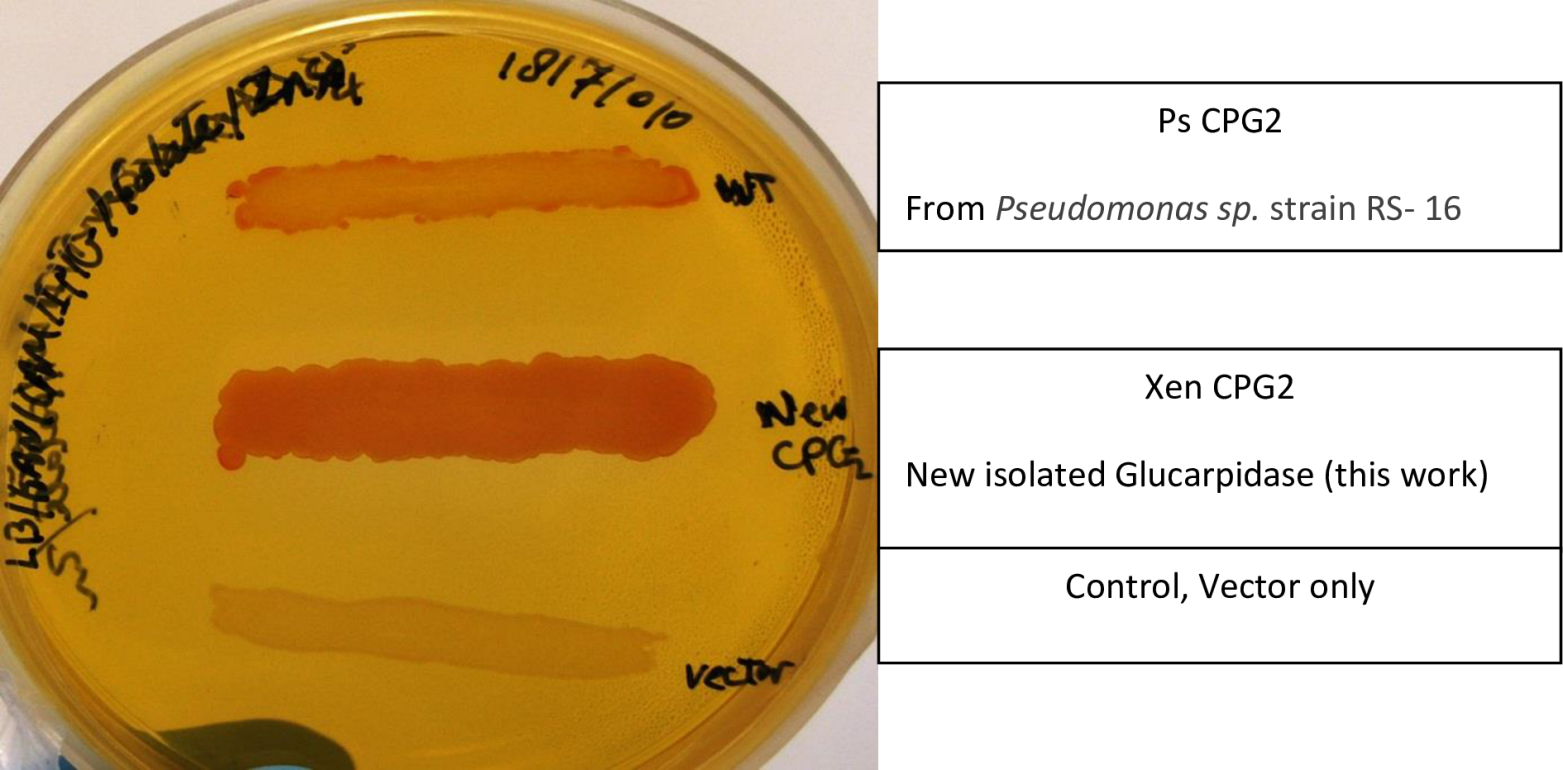
Recombinant CPG2 activity on folate agar plate. The activity of the isolated recombinant CPG2 in comparison to Ps CPG2 of *Pseudomonas sp* strain RS-16 and in the presence of negative control (vector only) on LB/KAN/CAM/IPTG/Folate in the presence of 0.2 mM ZnSO_4_.

The isolated recombinant Xen CPG2 showed high glucarpidase activity toward folate degradation on agar plates (Fig 6) in comparison to the Ps CPG2 recombinant enzyme in a negative control (empty vector). Therefore, we isolated the recombinant glucarpidases to test their activity *in vitro*.

**Fig 7:**
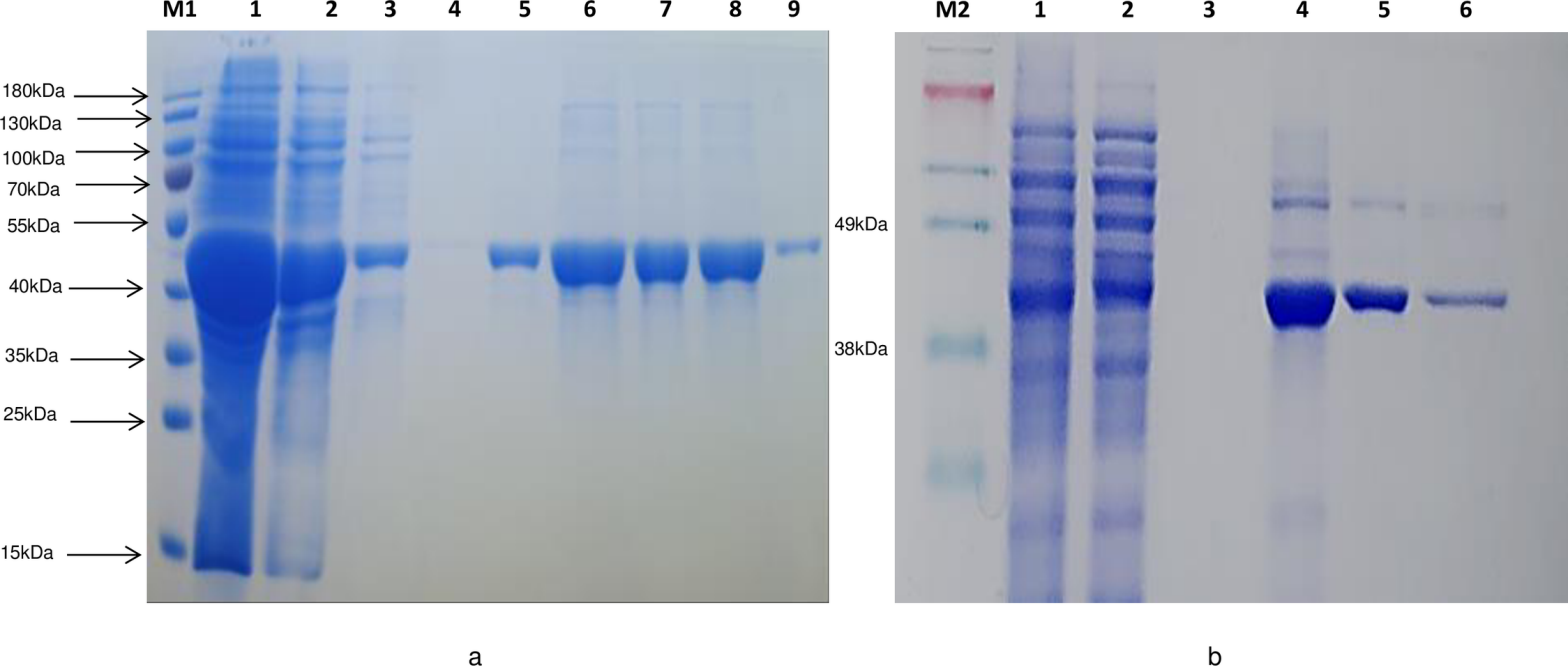
Purification of recombinant new glucarpidase relative to Ps CPG2. Coomassie blue staining of a 10% SDS-PAGE gel. M; Size markers in kiloDaltons; M1 is PageRuler Prestained Protein Ladder (10 to 180 kDa) and M2 is SeeBlue Plus prestained standard (↱3 to 198 kDa). **a)** Xen CPG2 purification; lane 1 is total soluble fraction of glucarpidase after centrifugation; 2, flow through; 3–4, wash 1 and wash 5; 5–9, eluted fractions from the Ni-NTA column with 400 mM imidazole. **b)** Ps CPG2 purification; lanes 1, 2, 3 are total, flow through, wash, lanes 4–6 are elution fractions.

We found that single step purification by Ni-affinity chromatography was sufficient to obtain highly pure Xen CPG2 (Fig 7A).

We also optimized the overexpression of the Ps CPG2 and purified the recombinant protein (Fig 7B).

**Fig 8:**
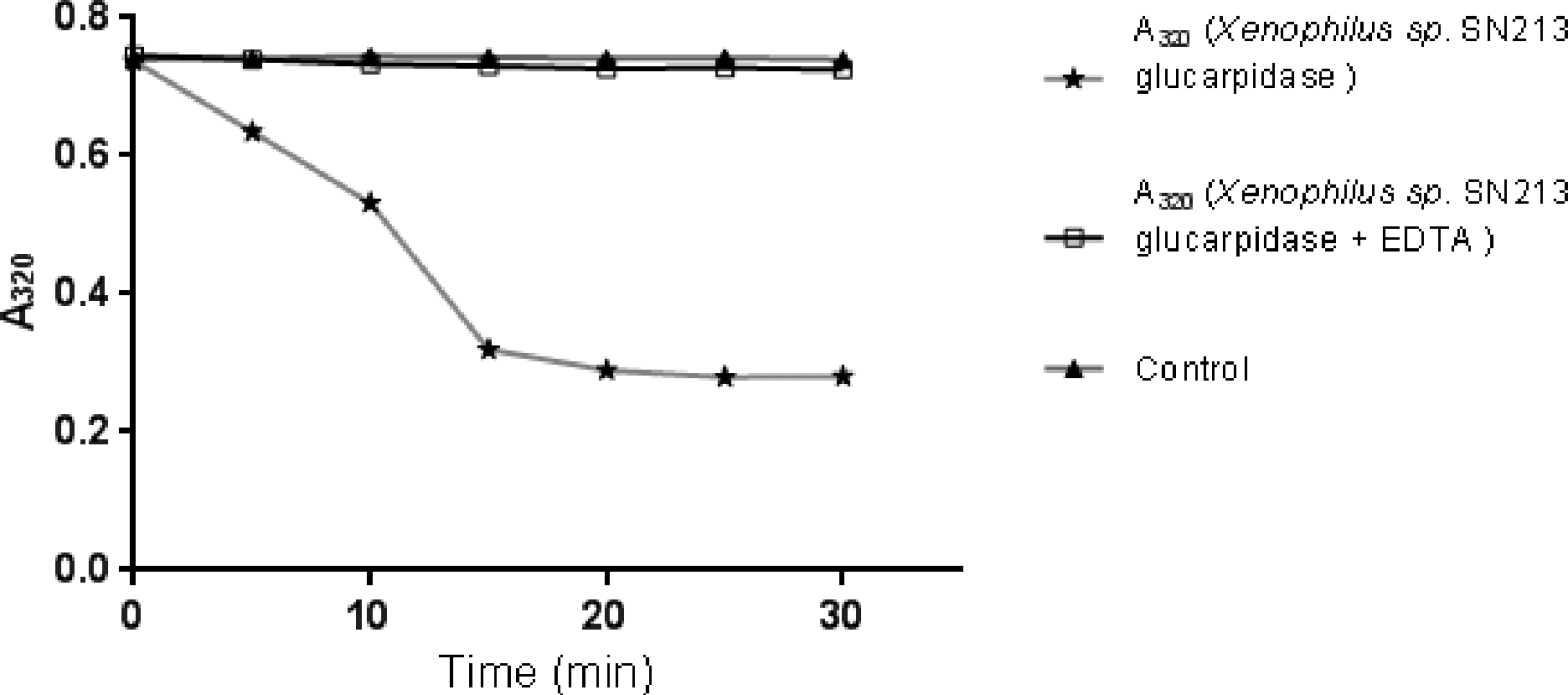
Carboxypeptidase G2 activity is Zn^^2+^^ dependent. MTX substrate solution and pure CPG2 in the presence of Zn^2+^ (star), control reaction of MTX substrate solution and buffer (filled triangle) and MTX substrate solution and pure CPG2 in the presence of Zn^2+^ and 10 mM EDTA (square) are shown. New isolated CPG2 is Zn^2+^ dependent.

The activity of the isolated enzyme on methotrexate was studied as shown in (Fig 8) and indicates that the isolated glucarpidases are metalloenzymes and require Zn^2+^ for activity.

### Kinetic properties of Xen CPG2 and Ps CPG2

Kinetic studies of the enzymes, Xen CPG2 and Ps CPG2 were carried out using a range of methotrexate substrate concentrations (0.03–0.42 mM) under standard assay conditions. Using the Lineweaver–Burk plot, the K_m_ and V_max_ values of 50.5 µM and 24.3 µM/min for Xen CPG2 and 171.7 µM and 52.6 µM/min for Ps CPG2 were obtained. The K_cat_ for both enzymes, Xen CPG2 and Ps CPG2 are 11.49 S^-1^ and 24.83 S^-1^. In addition, the specific activities of Xen CPG2 and Ps CPG2 were determined to be 28.84 nmol/min/mg and 62.6 nmol/min/mg, respectively.

### Circular dichroism (CD) spectral analysis

**Fig 9:**
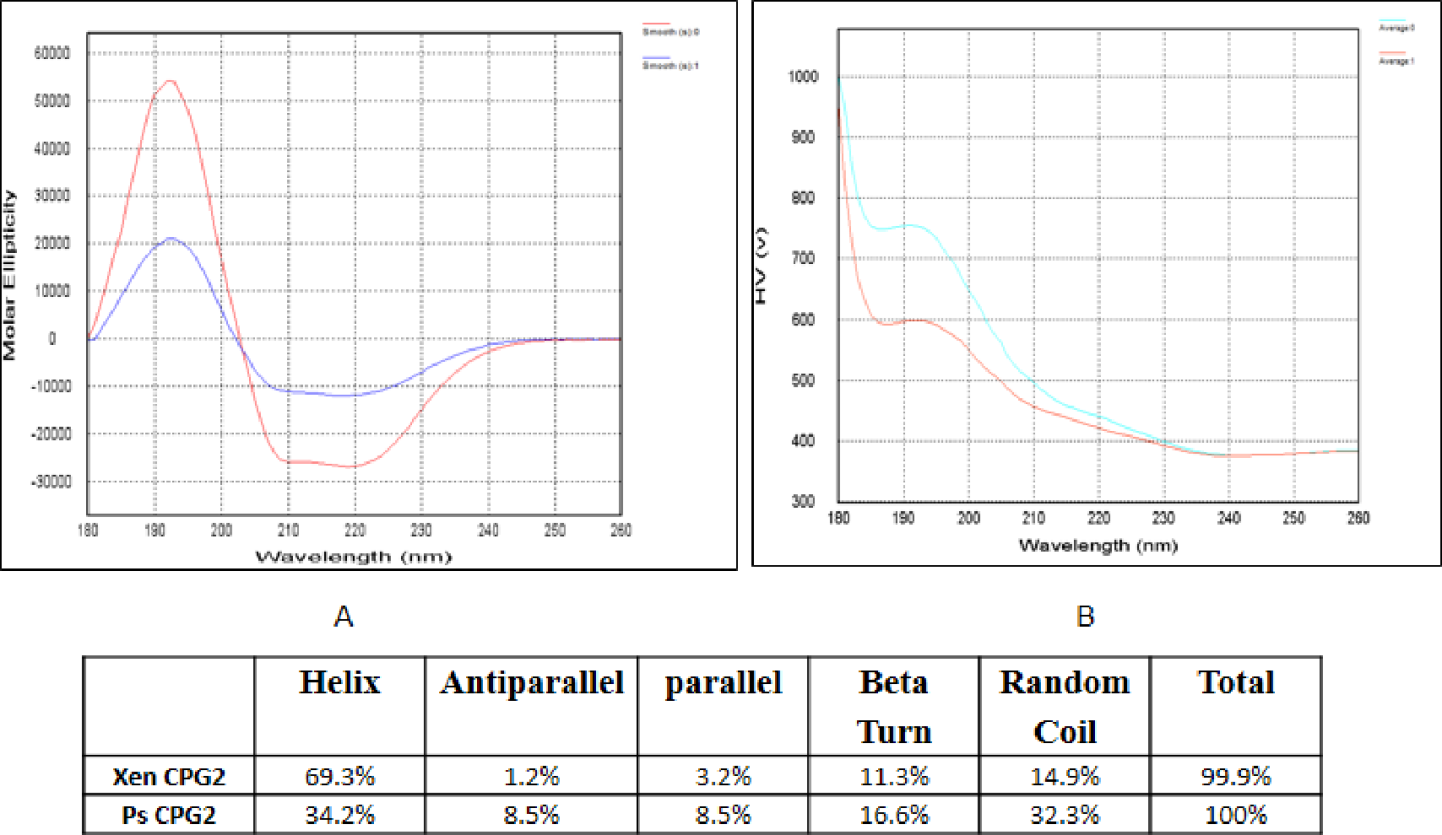
CD spectra and high voltage of Xen CPG2 and Ps CPG2. A) Combined CD spectra data of Xen CPG2 and Ps CPG2 in molar ellipticity relative the wavelength in far UV region, the spectra obtained by dragging their spectral data over each other, where smooth 0 is molar ellipticity of Xen CPG2 and smooth 1 is for CD spectra of Ps CPG2. All Spectral data are corrected for the baseline buffer, B) represents the combined High voltage (HV) for both enzymes where average 0 is Xen CPG2 and average 1 is Ps CPG2. Also the table shows the calculated protein secondary structure of Xen CPG2 and Ps CPG2 by CDNN deconvolution analysis using their CD spectral data.

The changes in the obtained CD magnitude (i.e. chirality of the signal, either positive or negative). In addition, the characteristic shape of the detected far UV CD signals for Ps CPG2 and Xen CPG2 (Fig 9) give insight about the extent to which they differed in the individual proteins' inter-molecular secondary structure. CD deconvolution results displaying the relative percentage of each secondary structure are shown in the attached table in Fig 9.

The percentage change of the four main secondary structures calculated by CDNN deconvolution analysis of the spectra is shown in Fig 9, that consists of 69.3% alpha helix, 1.2% antiparallel, 3.2% parallel, 11.3% beta turn, and 14.9% random coil for Xen CPG2, and 34.2% helix, 8.5% antiparallel, 8.5% parallel, 16.6% beta turn, and 32.3% random coil for Ps CPG2.

### Homology modeling of Xen CPG2

**Fig 10:**
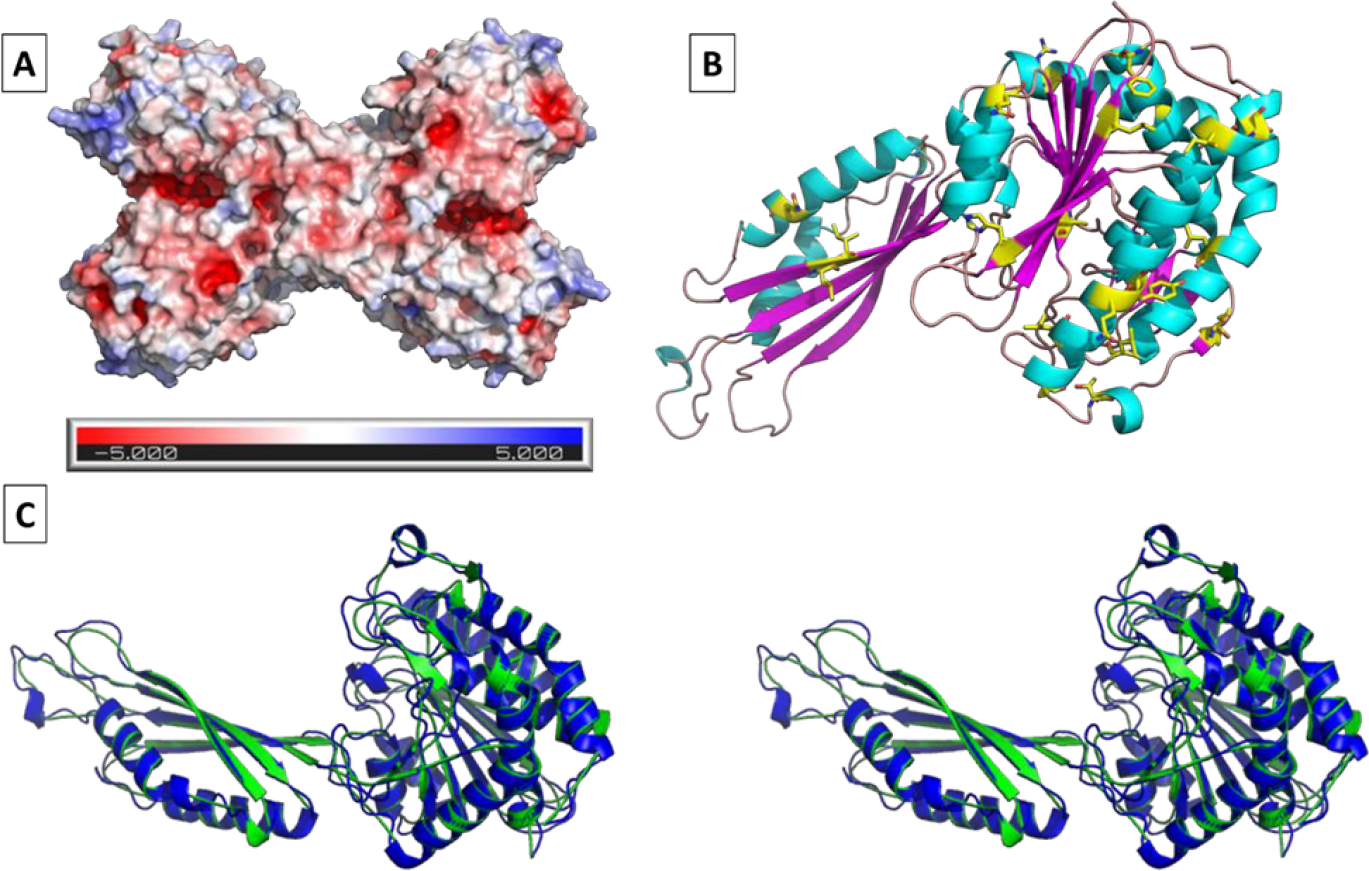
Homology modeling of Xen CPG2. **A.** Electrostatic surface presentation of the Xen CPG2 tetramer. Positive and negative charges are shown in blue and red, respectively. **B.** Color rendering of a Xen CPG2 monomer. Helix in cyan, b-sheet in pink, loops in brown and amino acids which differ from the model carboxypeptidase G2 are shown as yellow sticks. Rendering was performed using PyMol. **C.** Stereoview of the alignment of CPG2 Pseudomonas sp. Strain RS-16 (blue cartoon, PDB ID 1CG2) with the model of Xen CPG2 (green cartoon, RMS = 0.084 (374 to 374 atoms)).

Homology modeling was performed to produce the X ray structure of the Xen CPG2 and the alignment with the Ps CPG2 is shown in Fig 10.

### Anti Xen CPG2 polyclonal antibody response to Ps CPG2

**Fig 11:**
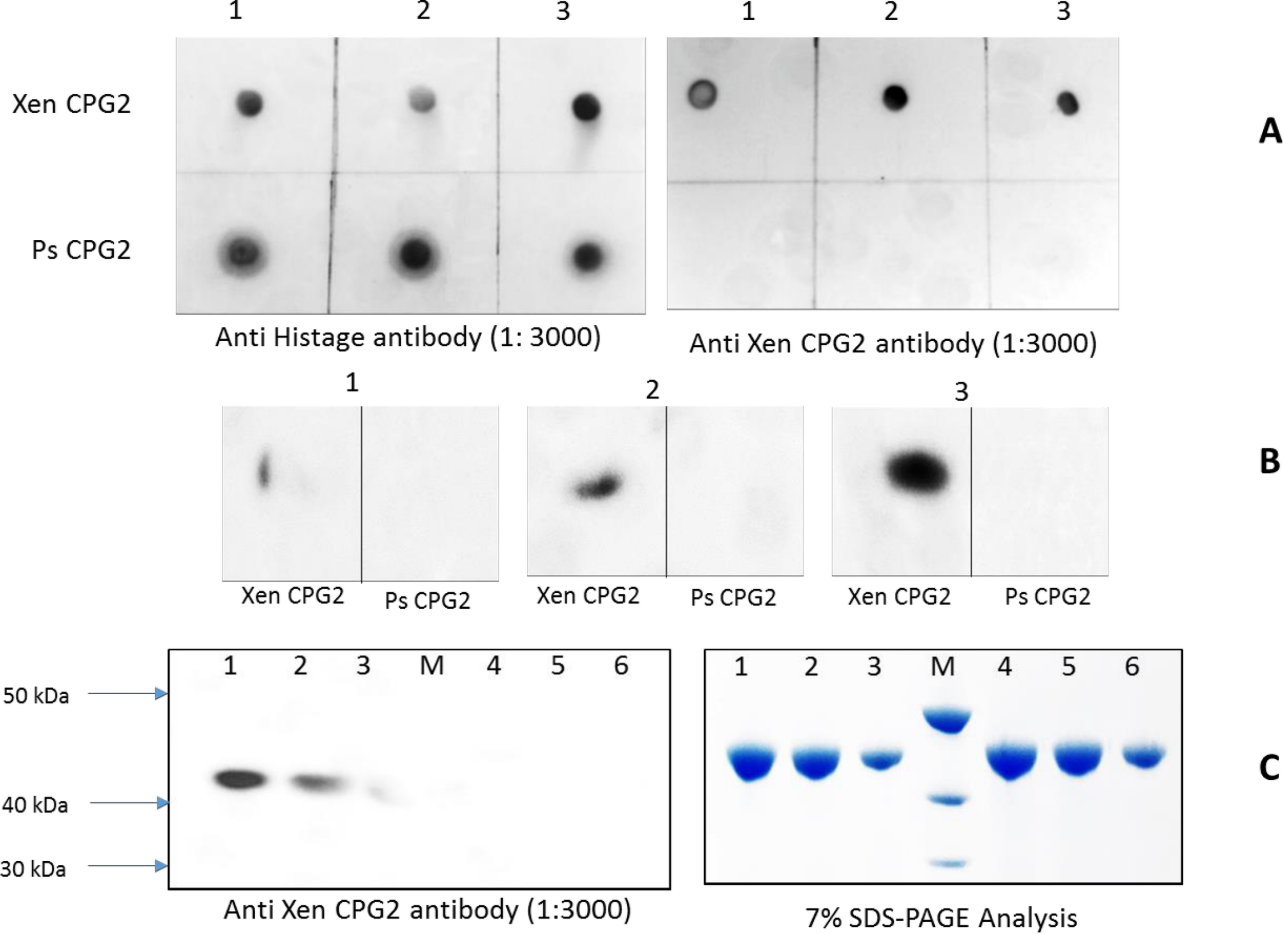
Antibody detection of newly isolated CPG2 relative to Ps CPG2. **A.** Dot blot using anti His tag antibody and using anti Xen CPG2 antibody where 1, 2, and 3 are pure protein (Xen CPG2 and Ps CPG2) at concentrations (0.05, 0.1, and 0.2 mg/mL). **B.** Dot blot at different concentration of anti Xen CPG2 antibody where 1, 2, and 3 are blotting at dilutions 1:20 000, 1:10 000 and 1:3000 in blocking buffer. **C.** SDS-PAGE and Western blot analysis of the pure protein (Xen CPG2 and Ps CPG2) where M is PageRuler™ Unstained Protein Ladder (10–200 kDa), lanes 1, 2, 3 are 0.25, 0.1, and 0.05 mg/mL of Xen CPG2 and lanes 4, 5, 6 are the same series of protein concentrations of Ps CPG2.

To test whether Ps CPG2, which is in current clinical use, was recognized by the antibody raised against the new CPG2 (Xen CPG2), we carried out dot blots and western blots on both enzymes (at different protein concentrations; 0.05, 0.1, and 0.2 mg/mL) using the Anti His tag antibody (positive control) and the Anti Xen CPG2 antibody as primary antibodies. In both cases (dot blot and Western Blot), the Anti His antibody bound to both CPG2s whereas the Anti Xen CPG2 bound strongly to the new CPG2 but showed little or no binding to the Ps CPG2 (Fig 11).

## Discussion

Targeted therapies have great advantages over the conventional chemotherapy and radiotherapy in significantly reducing systemic toxicity, and severe side effects. To be effective, targeted therapy requires repeated cycles of administration of the protein drug. Such repeated cycles, however, will lead to the development of an antibody response which undermines the efficacy of the therapy. The antibody response development against a protein eventually involves the differentiation of the B-cell with antibody receptor with binding capacity to the epitopes on the protein therapeutics.

A number of strategies have been pursued to overcome the antibody response by modifying the therapeutic protein to minimize its immunogenicity [31, 32]. However, the modification of a protein therapeutic to avoid or minimize the immune response can be laborious and is sometimes difficult to achieve.

We propose in this study a different strategy to overcome the immunogenicity problem of CPG2 in cancer therapy. CPG2 can be used in cancer treatment in two ways: firstly, in rescue therapy to remove any overdose of methotrexate by the hydrolysis of methotrexate to the less harmful product. Secondly, in targeted cancer treatment, by coupling CPG2 to antibodies specific for tumor cells, and the use of a pro-drug that would be activated by the enzyme in the vicinity of the cancer. Both applications utilize the ability of CPG2 to cleave C-terminal glutamate moieties. The use of CPG2 in antibody directed enzyme pro-drug therapy and detoxification of methotrexate is hampered by the host immune system response against CPG2. The detoxification process will also benefit greatly from variants with higher specific activity than that currently in use.

We propose that the unwanted immunogenicity towards the CPG2 in the ADEPT technique could be circumvented by using different CPG2s sharing the same function but with different epitopes. These CPG2s could be used consecutively, and therefore any antibody produced in response to the previously used CPG2 will not neutralize a subsequent CPG2 with disrupted or different epitope(s), nor will it provoke an undesired immune reaction in the patient.

Because the CPG2 is a bacterial enzyme, we propose that screening for new and different CPG2 producing bacteria from soil, and isolation and cloning of the CPG2 might lead to a new CPG2 with different epitope(s).

The identification of the newly isolated strains was carried out by biochemical and the *16s rRNA* analysis. The *16s rRNA* analysis showed that the first strain shares 98% DNA homology with a strain *Xenophilus azovorans* and 97% homology with *Variovorax paradoxus*. From these results, it was evident that strain SN1 is a new strain and may belong to the family *Comamonadaceae*. We could not find any strain with identical *16s RNA* DNA sequencing. We, therefore, named this isolated strain as *Xenophilus azovorans* SN213 and the novel *16s RNA* was deposited in the GeneBank database with accession number EU650684. *16s rRNA* DNA analysis of the second strain indicates that the strain SF3 is a *Pseudomonas* species, therefore its named was proposed to be *Pseudomonas lubricans* strain SF168 and was deposited with FJ600733 accession number in the GeneBank database. The *16s rRNA* DNA analysis shows that the third strain has 100% homology with *Stenotrophomonas sp*. AB1 and therefore the third new strain was named *Stenotrophomonas sp*. SA.

Glucarpidase is Zn^2+^-dependent enzyme [12, 33]. We demonstrated that the isolated new strains are glucarpidase producers by showing that their protein extracts degrade methotrexate and also that the activity is Zn^2+^ dependent (Fig 3). After the successful isolation and the confirmation of three CPG2 producers, we embarked on the isolation, cloning and molecular characterization of the genes from two of these CPG2 isolated strains, the gene from the *Xenophilus azovorans* SN213 (*Xen CPG2*) and the *Stenotrophomonas sp* SA (*Sten CPG2*). Molecular characterization of the two new glucarpidases, Xen CPG2 and Sten CPG2, revealed that Xen CPG2 encode a putative peptide of 415 amino acids, with about 94% similarity to the corresponding polypeptides of the *Pseudomonas sp*. Strain RS-16 glucarpidase (Fig 4) [12] which is currently in clinical use.

The amino acid sequence of Sten CPG2 shows a difference of three amino acids with the Xen CPG2. The predicted molecular weights of each recombinant protein are ≈ 41.761 kDa and 41.696 kDa for Xen CPG2 and Sten CPG2, respectively. These values are similar to those of other glucarpidases [11, 12].

The two new glucarpidases were cloned and overexpressed, in a soluble form exceeding 40% of the total soluble protein. The overexpression of the Xen CPG2 is shown in Fig 5. Due to the high expression of the CPG2 in the soluble form, we carried out all our studies on the soluble part and we did not perform a refolding process of the inclusion bodies. The new CPG2 (Xen CPG2) exerts higher activity towards folate hydrolysis on folate agar plate than Ps CPG2 (Fig 6), indicating that either the new CPG2 is much more active than Ps CPG2, or the Xen CPG2 was expressed in more soluble form than the Ps CPG2. The kinetic studies are consistent with the later explanation that the Xen CPG2 was expressed in a soluble form more than the Ps CPG2. At the same time, its pure recombinant protein (Fig 7A) confirmed that Xen CPG2 is a Zn^2+^ dependent metalloenzyme [34] as it showed maximum hydrolytic activity for MTX in the presence of Zn^2+^ ion that completely depleted upon chelation by addition of EDTA (Fig 8).

We also managed to optimise the condition to overexpress, in soluble form, the codon optimized Ps CPG2 in *E*. *coli* [28] (Fig 7B).To confirm that both enzymes hydrolyse folate in the same way the MS analysis of the insoluble materials formed from the folate degradation by the clones carrying Xen CPG2 and Ps CPG2 was carried out. Fig 2B and 2C shows the formation of DAMPA in both clones, with mass at 313.1m/z which consistent with the calculated value.

The soluble active recombinant Ps CPG2 we obtained in this study was used to investigate whether it is detected by a polyclonal antibody raised against Xen CPG2 and also to carry out a comparison study on both CPG2s: Xen CPG2 and Ps CPG2.

To shed some light on the secondary structures of both enzymes CD spectroscopy analysis was carried out. The change in secondary structure components as calculated by the deconvolution analysis software of CD spectra of Xen CPG2 and Ps CPG2 showed a significant increase in the alpha helix calculated component rather than the other secondary structure components, namely β-sheet (both parallel and anti-parallel), β-turn, and random coil of Xen CPG2 (69.3%) relative to Ps CPG2 (34.2%) indicating that Xen CPG2 has more folding structure than that of Ps CPG2 (Fig 9).

We also predicted the structure of Xen CPG2 based on its sequence (Fig 10). The best model based on the primary structure of Xen CPG2 we found was carboxypeptidase G2 (PDB ID 1CG2) with a sequence homology of 94%. This was used as a template to predict the tertiary and quaternary structure of Xen CPG2 (Fig 10A and 10B). We also did the structural alignment of the predicted structure of Xen CPG2 with CPG2 from *Pseudomonas sp*. strain RS-16. (PDB ID 1CG2) [30]. Not surprisingly we found an overall very good structural match with an RSM = 0.084. The only significant changes of the alignment were found in the flexible loop, C- and N-terminal regions (Fig 10C).

The kinetic studies of the two CPG2s showed that at the same protein concentration Xen CPG2 has lower K_m_ value (50 µM) in comparison to Ps CPG2 (170 µM), indicating its higher affinity towards MTX than Ps CPG2 and although Xen CPG2 shows a lower turnover number K_cat_ (11.49 S^-1^) than Ps CPG2 (24.83 S^-1^), Xen CPG2 shows about double the kinetic efficiency (kinetic perfection) of Ps CPG2 based on their calculated values K_cat_/K_m_.

The strategy of mutating B-cell epitopes to reduce immunogenicity has also been implemented successfully [35–37] and promises to be a useful technique that has a wide range of applications for recombinant cancer therapy and other diseases. The work presented here, however, to the best of our knowledge, is the first to report upon the characterization of two different but related protein therapeutics with the same function but with different epitope structures. Although the data reported here (Fig 11) needs to be confirmed using blood sera from patients treated with the Ps CPG2, it strongly suggests that the two enzymes are likely to have different epitopes and hence may be of clinical value.

Methotrexate is a potent and effective therapeutic drug, not only in cancer therapy, but also in rheumatoid arthritis (RA), diabetes mellitus, and other inflammatory diseases. The availability of new glucarpidases could be of great importance in improving its therapeutic usefulness in cancer therapy and will provide the opportunity for dose studies that, in turn, might lead to the escalation of methotrexate doses for more efficient treatment.

## Supporting information

S1 Fig Phylogenetic positions of the isolated strain *Xenophilus azovorans* SN213. This was achieved by using Fast Minimum Evolution Tree Method (NCBI) based on the 16s rRNA sequences of *Xenophilus sp*. *SN213* and some other related taxa. Scale bar represents 0.004 nucleotide substitutions per site.
(PDF)

S2 Fig Phylogenetic positions of the isolated strain *Pseudomonas lubricans* strain SF168. This was achieved by using Fast Minimum Evolution Tree Method (NCBI) based on the 16s rRNA sequences of *Pseudomonas oleovorans* species and some other related taxa. Scale bar represents 0.0002 nucleotide.
(PDF)
