# Correction: Isolation and molecular characterization of novel glucarpidases: Enzymes to improve the antibody directed enzyme pro-drug therapy for cancer treatment

**DOI:** 10.1371/journal.pone.0294885

**Published:** 2023-11-21

**Authors:** Fatma B. Rashidi, Alanod D. Al-Qahtani, Sara S. Bashraheel, Shabnam Shaabani, Matthew R. Groves, Alexander Dömling, Sayed K. Goda

The Data Availability statement in [1] is incorrect as all relevant data were not provided within the article and supporting information files. With this notice, the authors provide the primary data for Figs 3, 8, 9 and 11 in S1–S4 Files.

There was a spelling error in the second author’s name. The correct name is: Alanod D. Al-Qahtani. The correct citation is: Rashidi FB, Al-Qahtani AD, Bashraheel SS, Shaabani S, Groves MR, Dömling A, et al. (2018) Isolation and molecular characterization of novel glucarpidases: Enzymes to improve the antibody directed enzyme pro-drug therapy for cancer treatment. PLoS ONE 13(4): e0196254. https://doi.org/10.1371/journal.pone.0196254

In addition, the authors wish to acknowledge that the image in Fig 7B of this article [1] was reused in Fig 1A of a later article from the same group [2]. These images present the same experimental condition, isolation of wild-type *Pseudomonas Putida* CPG2 (Ps CPG2). Although the corresponding author referenced the original source [1] in the later article [2], he acknowledges that it should have also been indicated in the figure legend of the article [2]. The *PLOS ONE* Editors have assessed this issue and have no concerns about the reuse of the image from [1].

## Supporting information

S1 File Quantitative data underlying Fig 3 and 8. (XLSX)

S2 File Original images underlying Fig 7. (ZIP)

S3 File CD Spectra and settings related to Fig 9. (ZIP)

S4 File Original images underlying Fig 11. (ZIP)

## References

[1] Rashidi FB, AlQhatani AD, Bashraheel SS, Shaabani S, Groves MR, Dömling A, et al. (2018) Isolation and molecular characterization of novel glucarpidases: Enzymes to improve the antibody directed enzyme pro-drug therapy for cancer treatment. PLoS ONE 13(4): e0196254. 10.1371/journal.pone.0196254 29698433 PMC5919439

[2] Al-Qahtani A. D., Bashraheel S. S., Rashidi F. B., O’Connor C. D., Romero A. R., Domling A., & Goda S. K. (2019). Production of “biobetter” variants of glucarpidase with enhanced enzyme activity. Biomedicine & Pharmacotherapy, 112, 108725. 10.1016/j.biopha.2019.108725 30970523

