# Supplementary figures and images for: Isolation and molecular characterization of novel glucarpidases: Enzymes to improve the antibody directed enzyme pro-drug therapy for cancer treatment
Source: PLoS One. 2018 Apr 26;13(4):e0196254. doi: 10.1371/journal.pone.0196254 (PMC5919439; doi:10.1371/journal.pone.0196254)

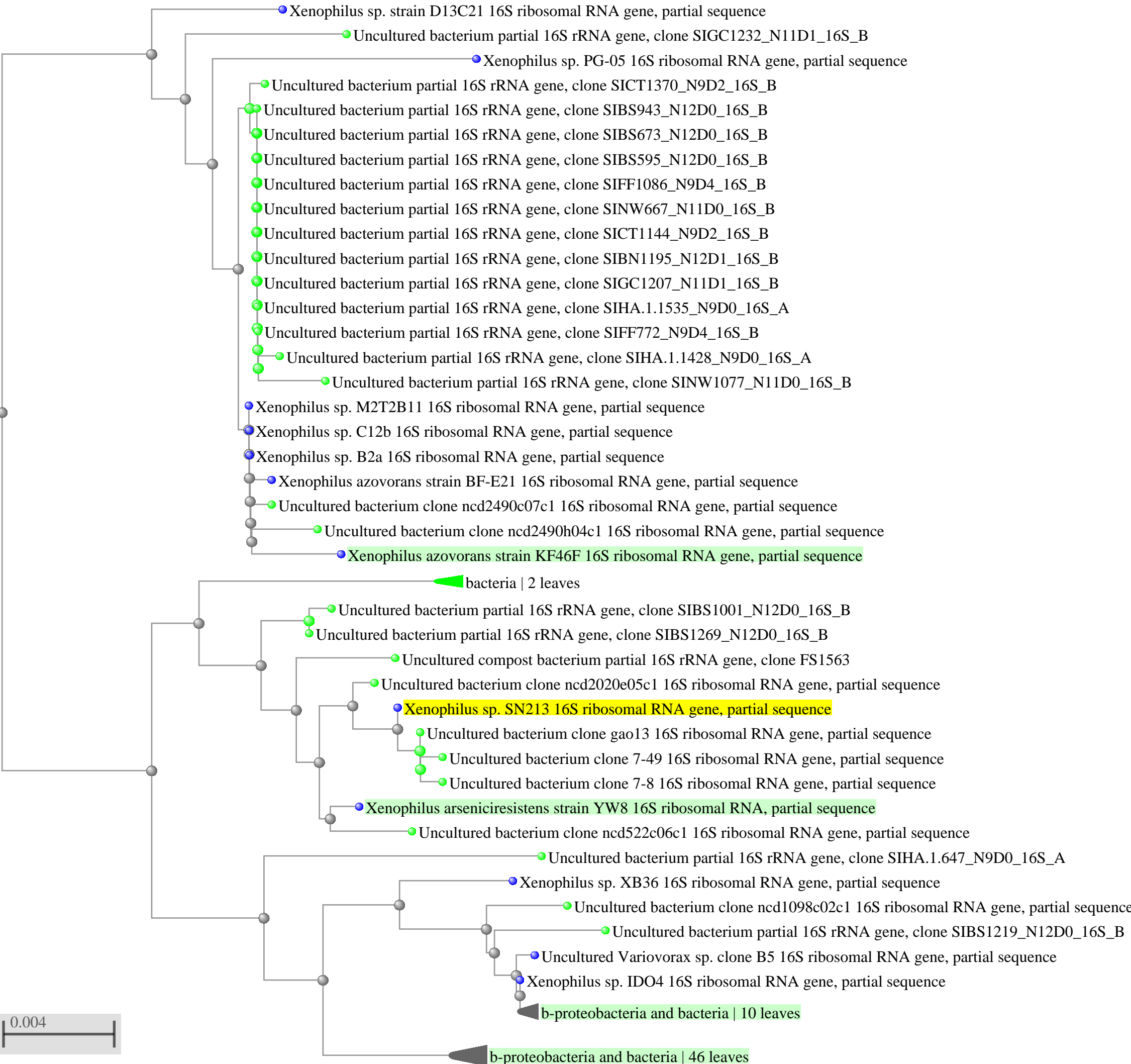

Supplement: S1 Fig — This was achieved by using Fast Minimum Evolution Tree Method (NCBI) based on the 16s rRNA sequences of Xenophilus sp. SN213 and some other related taxa. Scale bar represents 0.004 nucleotide substitutions per site. (PDF) [file pone.0196254.s001.pdf]

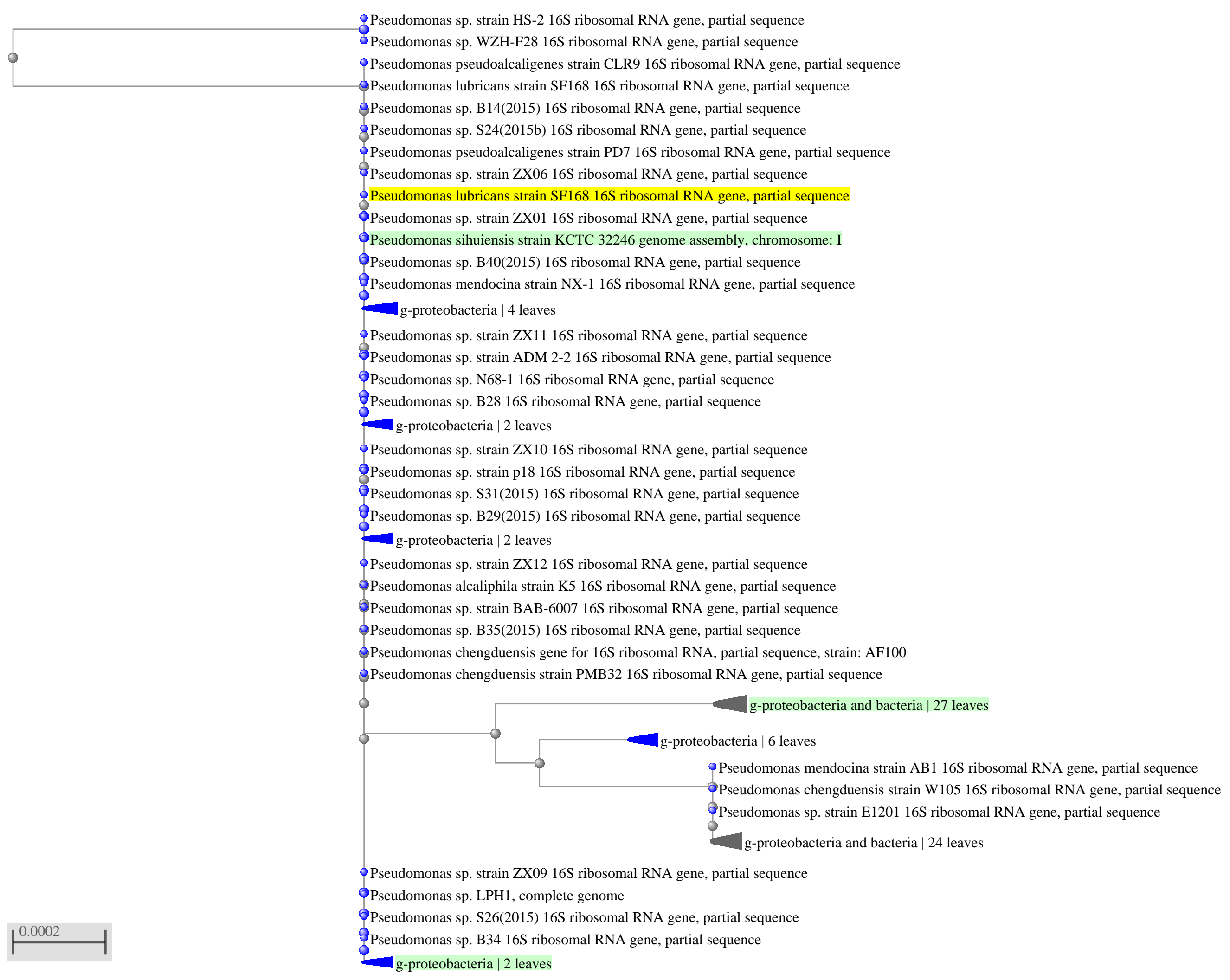

Supplement: S2 Fig — This was achieved by using Fast Minimum Evolution Tree Method (NCBI) based on the 16s rRNA sequences of Pseudomonas oleovorans species and some other related taxa. Scale bar represents 0.0002 nucleotide. (PDF) [file pone.0196254.s002.pdf]
