# Supplementary figures and images for: Correction: Isolation and molecular characterization of novel glucarpidases: Enzymes to improve the antibody directed enzyme pro-drug therapy for cancer treatment
Source: PLoS One. 2023 Nov 21;18(11):e0294885. doi: 10.1371/journal.pone.0294885 (PMC10662713; doi:10.1371/journal.pone.0294885)

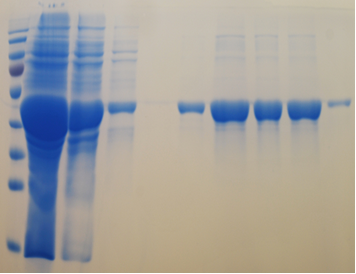

Supplement: S2 File — (ZIP) [file pone.0294885.s002.zip › S2 File. Original images for Fig 7/Fig 7a.png]

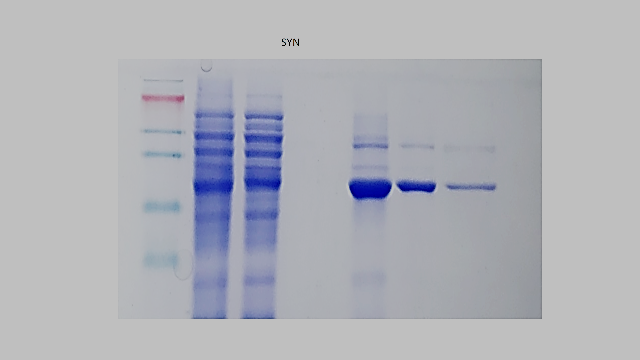

Supplement: S2 File — (ZIP) [file pone.0294885.s002.zip › S2 File. Original images for Fig 7/Fig7b.png]

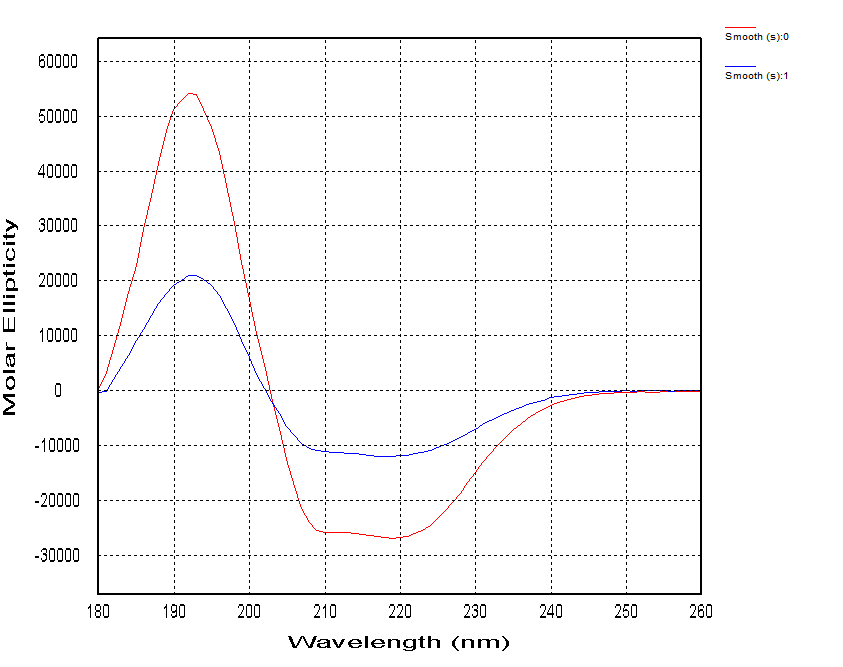

Supplement: S3 File — (ZIP) [file pone.0294885.s003.zip › S3 File. Spectra and settings for Fig 9/azo syn molar ellepticity Fig 9A.png]

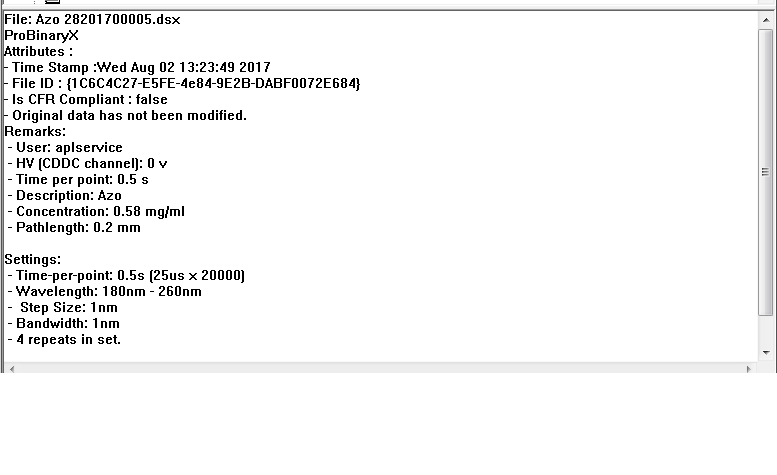

Supplement: S3 File — (ZIP) [file pone.0294885.s003.zip › S3 File. Spectra and settings for Fig 9/CD informatrion setting related to Fig 9.png]

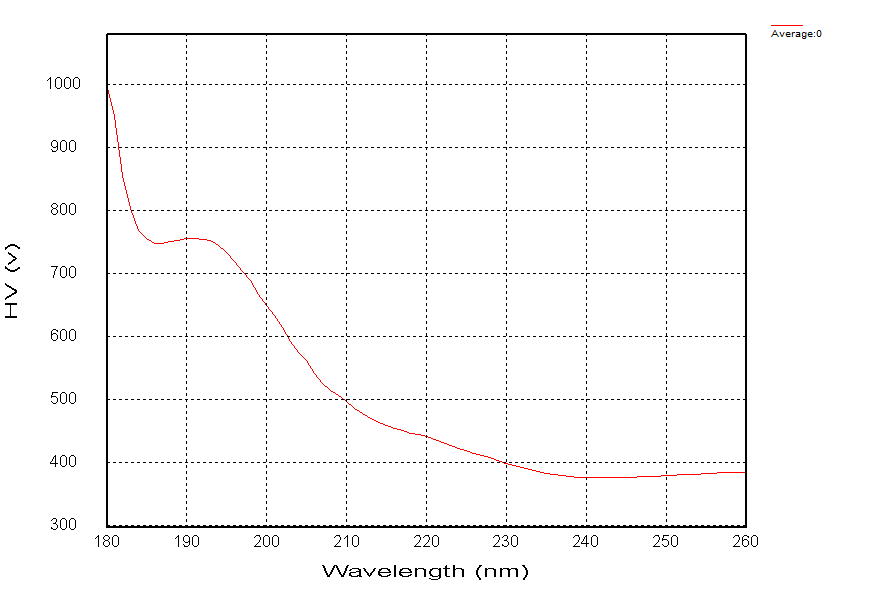

Supplement: S3 File — (ZIP) [file pone.0294885.s003.zip › S3 File. Spectra and settings for Fig 9/HV azo related to figur 9.png]

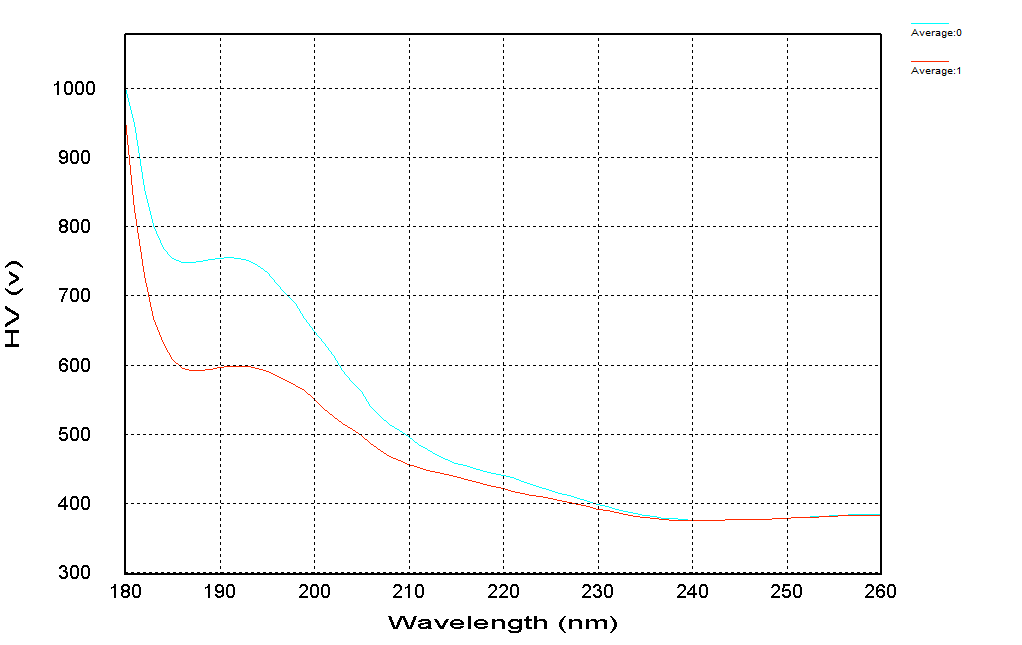

Supplement: S3 File — (ZIP) [file pone.0294885.s003.zip › S3 File. Spectra and settings for Fig 9/HV combined of syn and Azo Fig 9 B.png]

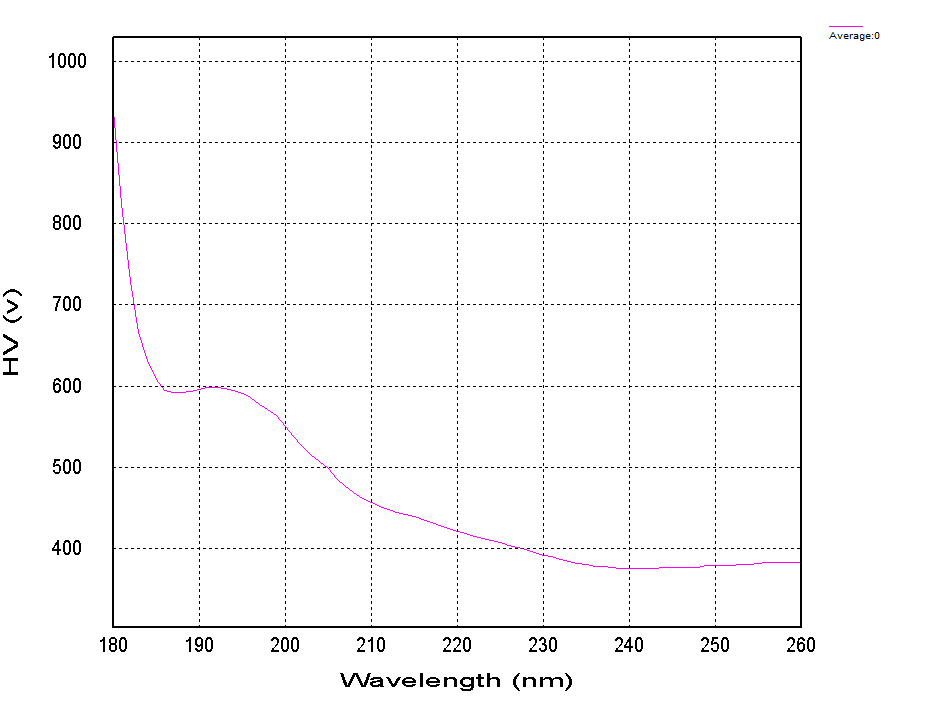

Supplement: S3 File — (ZIP) [file pone.0294885.s003.zip › S3 File. Spectra and settings for Fig 9/HV syn related to fig 9.png]

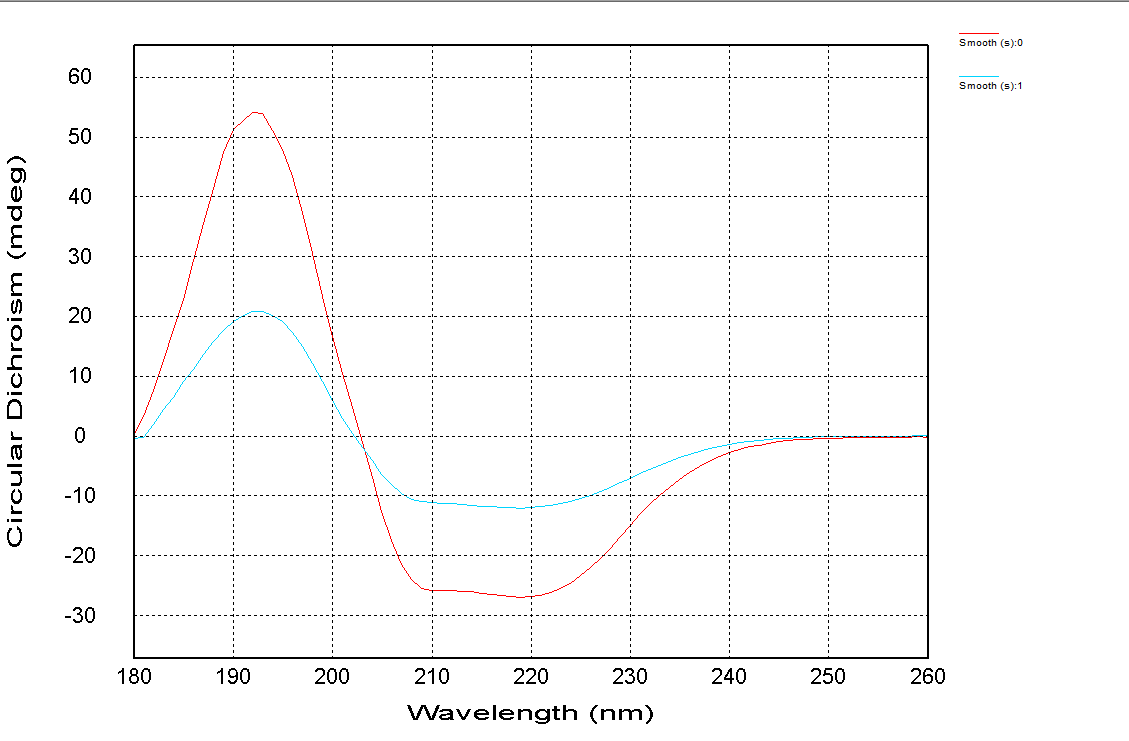

Supplement: S3 File — (ZIP) [file pone.0294885.s003.zip › S3 File. Spectra and settings for Fig 9/related to Fig 9.png]

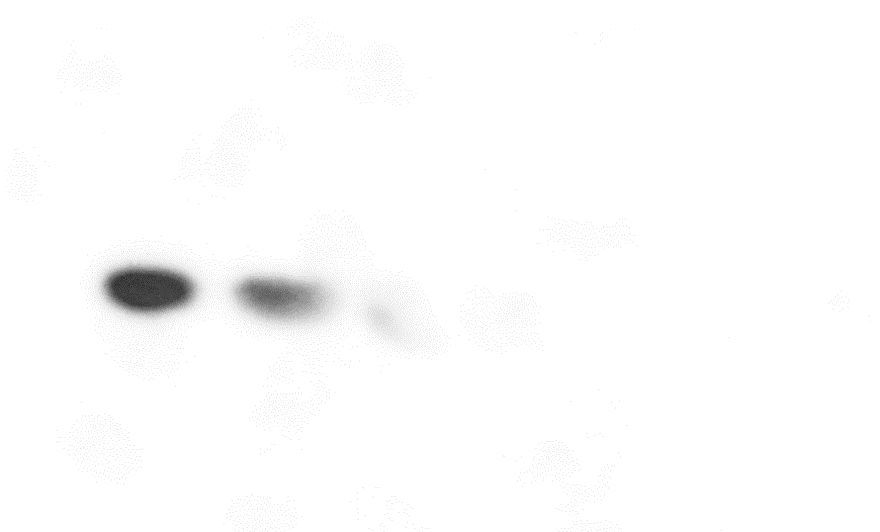

Supplement: S4 File — (ZIP) [file pone.0294885.s004.zip › S4 File. Original images for Fig 11/Fig 11c WB.png]

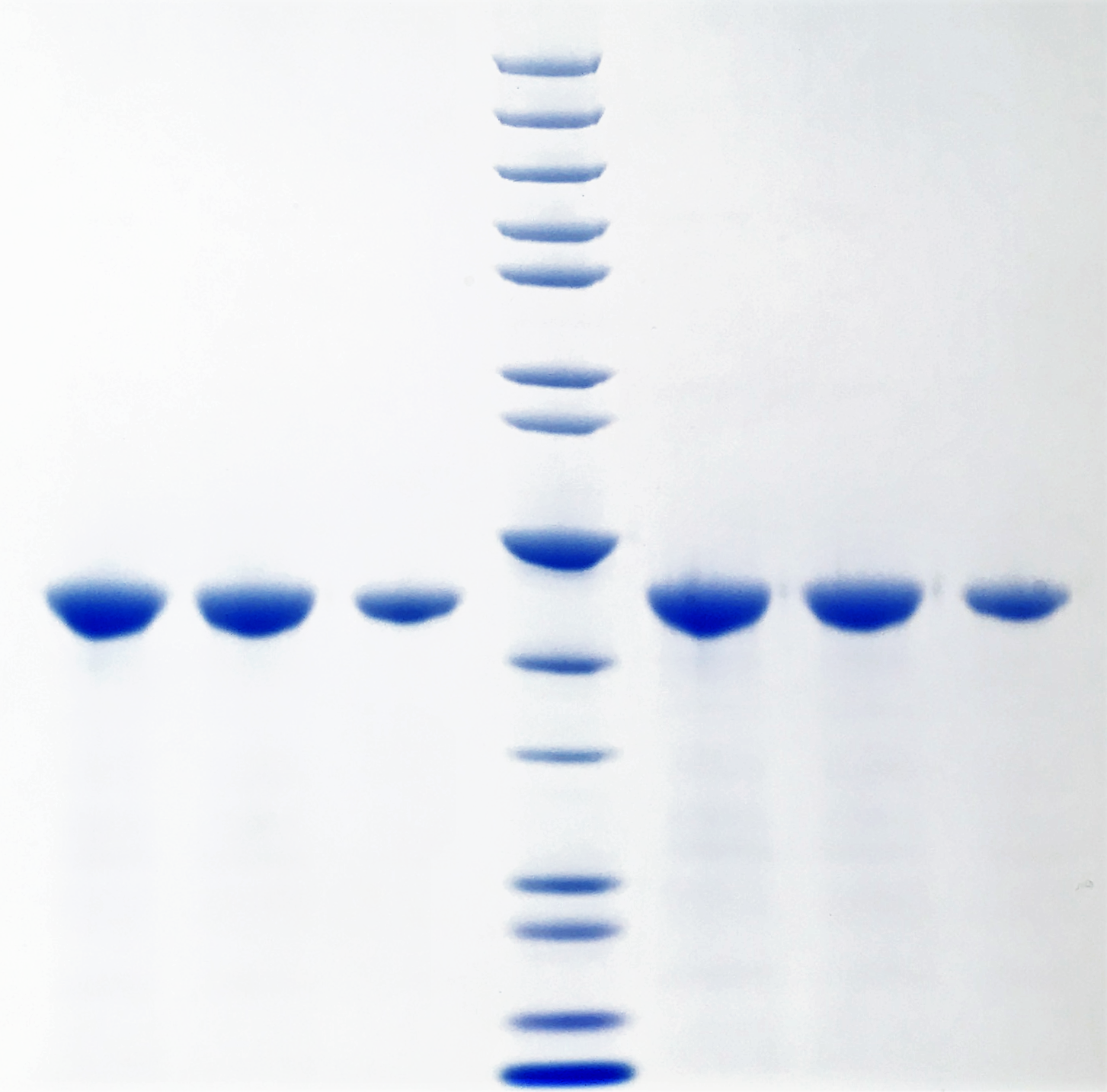

Supplement: S4 File — (ZIP) [file pone.0294885.s004.zip › S4 File. Original images for Fig 11/Fig11c SDS.png]
